# Engineering Artificial Mitochondria with Self‐Amplifying Proton Generation for Autonomous Energy Supply and Metabolic Coupling in Artificial Cells

**DOI:** 10.1002/anie.202514980

**Published:** 2025-09-30

**Authors:** Fangqin Fu, Xuemei Hu, Shijia Tao, Yu Gao, Daniel Crespy, Katharina Landfester, Xiangzhao Mao, Shuai Jiang

**Affiliations:** ^1^ Key Laboratory of Marine Drugs Chinese Ministry of Education School of Medicine and Pharmacy Ocean University of China Qingdao 266003 P.R. China; ^2^ State Key Laboratory of Marine Food Processing and Safety Control Ocean University of China Qingdao 266404 P.R. China; ^3^ Laboratory for Marine Drugs and Bioproducts Qingdao Marine Science and Technology Center Qingdao 266237 P.R. China; ^4^ Department of Materials Science and Engineering School of Molecular Science and Engineering Vidyasirimedhi Institute of Science and Technology (VISTEC) Rayong 21210 Thailand; ^5^ Max Planck Institute for Polymer Research Ackermannweg 10 55128 Mainz Germany; ^6^ College of Food Science and Engineering Ocean University of China Qingdao 266003 P.R. China

**Keywords:** Bioenergy, Cascade reactions, Metabolic mimicry, Mitochondria, Oxidative phosphorylation

## Abstract

A continuous and autonomous energy supply is essential for sustaining life‐like biochemical processes in artificial cells. Although considerable efforts have been devoted to engineering artificial organelles that emulate mitochondrial energy conversion, the generation of a robust transmembrane proton gradient—essential for driving efficient ATP production—remains a major challenge. Here, we present a mitochondria‐mimicking ATP nano‐generator constructed through quantitative co‐compartmentalization of glucose oxidase and catalase within silica nanocapsules. Enzymes are encapsulated in situ during the formation of core‐shell nanocapsules, enabling precise loading, effective protection, and creation of a confined nanoscale reaction chamber that fosters catalytic synergy. Within this microenvironment, catalase rapidly decomposes H_2_O_2_ to generate O_2_, which is in turn utilized by glucose oxidase—thus establishing a self‐reinforcing enzymatic cascade that amplifies proton production. After coating the enzyme‐loaded nanocapsules with an ATPase‐integrated liposome bilayer to construct the artificial mitochondrion, the resulting proton gradient across the membrane efficiently drives ATP synthase rotation, enabling high‐yield ATP production. When integrated into giant unilamellar vesicles (GUVs) as synthetic cell models, this system supports autonomous nicotinamide adenine dinucleotide (NADH) biosynthesis and glucose‐powered oxidative phosphorylation, mimicking key metabolic features of living mitochondria. This work establishes an effective and versatile platform for engineering energy‐autonomous artificial living systems, advancing the state of the art of bottom‐up synthetic biology.

## Graphical abstract

A mitochondria‐mimicking ATP generator improves the enzyme instability of enzyme‐based artificial mitochondria and eliminates the toxicity of H_2_O_2_ via quantitative enzyme co‐compartmentalization. The resulting artificial mitochondria can establish a sustained proton gradient for ATP synthesis. The integration of artificial mitochondria into artificial cells can enable glucose‐fueled ATP generation, powering an autonomous NADH biosynthesis.

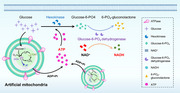

## Introduction

Internal processes in natural cells are autonomously coordinated through the precise assembly of diverse functional modules, enabling the execution of complex biological activities. Energy‐supplying modules, such as adenosine triphosphate (ATP)‐producing mitochondria, are essential for sustaining cellular metabolism.^[^ ^1^, ^2^ ^]^ Considerable efforts have been devoted to replicating the structures and functions of natural cells using bottom‐up strategies to construct artificial cell analogues.^[^ ^3^, ^4^, ^5^, ^6^ ^]^ These efforts have largely advanced our understanding of the intricate metabolic processes in living organisms,^[^ ^7^, ^8^, ^9^ ^]^ and have also led to the development of artificial systems that mimic cellular structure and functions. Representative examples include polymer vesicles^[^ ^10^ ^]^ and capsules,^[^ ^11^ ^]^ liposomes,^[^ ^12^ ^]^ virus‐like particles,^[^ ^13^ ^]^ inorganic cages,^[^ ^14^ ^]^ hydrogels,^[^ ^15^ ^]^ sponge‐like materials,^[^ ^16^ ^]^ and coacervates.^[^ ^17^ ^]^ Given the central role of ATP as the cellular energy currency, the continuous supply of ATP is essential for constructing functional artificial cells. However, achieving energy self‐sustainability in artificial cells remains a major challenge, primarily due to the complexity of constructing and integrating artificial ATP‐generating modules that can efficiently and stably replicate the functions of mitochondria. Overcoming this barrier is a critical prerequisite for developing truly autonomous artificial living systems capable of executing more sophisticated cellular functions and behaviors.

To this aim, recent research has focused on constructing artificial organelles that mimic natural bioenergetics. Central to these designs is the establishment of a proton motive force (PMF) across membranes and its coupling to ATP synthase (ATPase) for ATP production.^[^ ^18^ ^]^ Reported strategies to generate PMF include acid–base transitions,^[^ ^19^ ^]^ photoactive catalysts,^[^ ^20^ ^]^ and light‐driven proton pumps such as bacteriorhodopsin and Photosystem II (PSII).^[^ ^21^, ^22^ ^]^ Acid–base transitions can create defined pH gradients—for instance, ATPase‐containing polyelectrolyte microcapsules produced ATP under conditions of external pH 4.6 and internal pH 8.8—yet the PMF dissipated rapidly as proton concentrations equilibrated.^[^ ^19^ ^]^ Photoactive systems offer dynamic control: semiconducting graphitic carbon nitride (g‐C_3_N_4_) nanosheets catalyze light‐driven glucose oxidation to release protons,^[^ ^20^ ^]^ while bacteriorhodopsin reconstituted with ATPase in vesicles enabled ATP‐dependent protein synthesis.^[^ ^21^ ^]^ PSII has also been integrated with ATPase proteoliposomes for light‐to‐ATP conversion from water, though its intrinsic instability limits long‐term efficiency.^[^ ^23^ ^]^ Alternatively, glucose oxidase (GOx)‐driven artificial mitochondria generate protons through glucose oxidation and sustain ATP synthesis for several hours.^[^ ^24^ ^]^ However, these designs are constrained by limited proton yield, enzyme instability, and accumulation of inhibitory byproducts (e.g., H_2_O_2_). More broadly, reliance on natural enzymes poses persistent challenges of stability and immobilization, which restrict their sustained performance.

**Figure 1:**
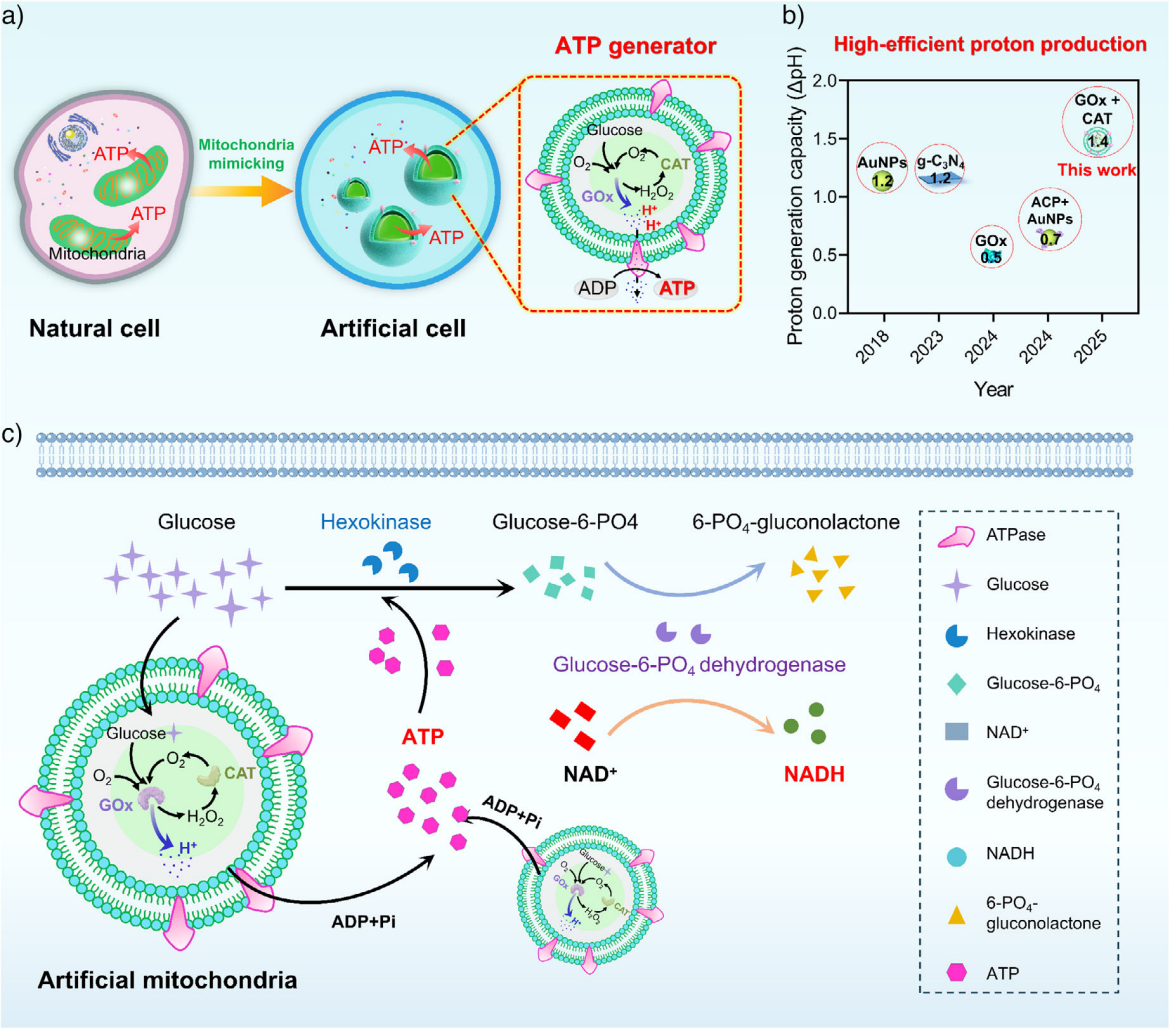
Schematic illustration of the construction of self‐energy‐supplying artificial cells and their application in NADH biosynthesis. a) Design of a mitochondria‐mimicking ATP generator. ATP production is driven by a proton gradient due to high‐yield proton generation within mesoporous silica nanocapsules. b) Proton generation capacity (ΔpH) of reported proton‐generating systems in the literature. AuNPs, gold nanoparticles^[^ ^27^ ^]^; g‐C_3_N_4_, graphitic carbon nitride^[^ ^20^ ^]^; GOx, glucose oxidase^[^ ^25^ ^]^; ACP, the natural acid phosphatase.^[^ ^26^ ^]^ c) NADH biosynthesis process powered by the artificial mitochondria. Hexokinase, glucose‐6‐PO_4_ dehydrogenase, and NAD^+^ were encapsulated within a giant vesicle. In this system, glucose was phosphorylated to glucose‐6‐PO_4_ by hexokinase in the presence of ATP. The glucose‐6‐PO_4_ was subsequently oxidized by glucose‐6‐PO_4_ dehydrogenase to reduce NAD^+^ to NADH.

In this study, we constructed an efficient mitochondria‐mimicking ATP generator, which enables an autonomous energy supply and metabolic coupling in artificial cells. In this system, GOx and catalase (CAT) were quantitatively co‐encapsulated at their optimized ratio in mesoporous silica nanocapsules (GC@SiO_2_). These nanocapsules were subsequently enveloped by liposomes, followed by the reconstitution of plant‐derived ATPase into the lipid bilayer, obtaining artificial mitochondria (Figure 1a). The spatial co‐encapsulation of CAT and GOx enhances enzymatic cascade efficiency by rapidly decomposing the toxic intermediate H_2_O_2_ into O_2_, which in turn fuels the GOx‐catalyzed reaction, thereby boosting proton production and establishing a robust PMF to drive ATP synthesis. Compared with previously reported proton‐pump‐based artificial mitochondria, such as GOx‐driven system (ΔpH ≈ 0.5),^[^ ^25^ ^]^ g‐C_3_N_4_ photoenzymes (ΔpH ≈ 1.2),^[^ ^20^ ^]^ and gold nanoparticles (AuNPs)‐natural acid phosphatase coupling system (ΔpH ≈ 0.7),^[^ ^26^ ^]^ our mitochondria‐mimicking ATP generator produces enhanced proton production (ΔpH ≈ 1.4) (Figure 1b). This stronger PMF directly accelerates ATPase rotation and thereby enhancing both the rate and efficiency of ADP phosphorylation. Notably, integration of the artificial mitochondria into giant unilamellar vesicles (GUVs) enables glucose‐fueled ATP production, which powers autonomous NADH biosynthesis and mimics oxidative phosphorylation within the artificial cells (Figure 1c).

## Results and Discussion

### Synthesis of the Proton Generator

**Figure 2:**
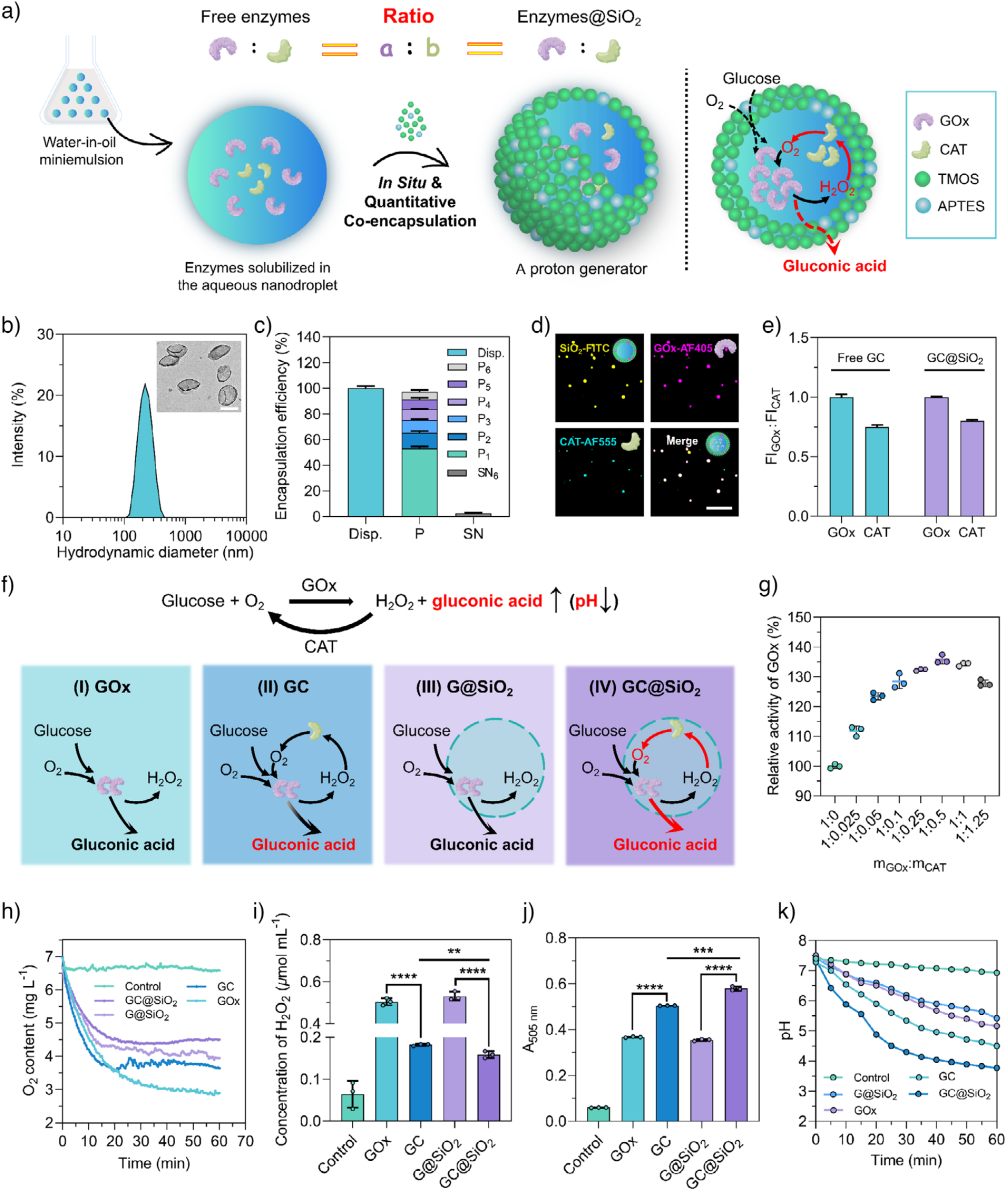
In situ quantitative co‐encapsulation of GOx and CAT in GC@SiO_2_ as a proton generator. a) Left: Schematic illustration of GC@SiO_2_ synthesis via an interfacial confined sol‐gel process in an inverse (water‐in‐oil) miniemulsion. Right: Mechanistic illustration of the dual‐enzyme cascade reaction within the system. b) Hydrodynamic diameter distribution of GC@SiO_2_, with insets showing TEM image of GC@SiO_2_. Scale bar: 200 nm. c) Encapsulation efficiency of enzymes in GC@SiO_2_. Disp.: original dispersion, P: precipitates collected after centrifugation, SN: supernatants from centrifugation (*n* = 3). d) CLSM images showing the overlap of fluorescent components in GC@SiO_2_: FITC‐labeled capsule (yellow, upper left), AF405‐labeled GOx (magenta, upper right), and AF555‐labeled CAT (cyan, lower left). Scale bar: 5 µm. e) Fluorescence intensity ratio between AF405‐labeled GOx and AF555‐labeled CAT in their free form versus encapsulated state within GC@SiO_2_ (*n* = 3). f) Schematic illustration of the catalytic mechanisms of single‐enzyme (GOx) and dual‐enzyme (GOx and CAT) systems before and after encapsulation within SiO_2_ nanocapsules: (I) unencapsulated GOx, (II) unencapsulated GOx and CAT, (III) GOx encapsulated in SiO_2_ nanocapsules, and (IV) GOx and CAT co‐encapsulated in SiO_2_ nanocapsules. The upper panel depicts the equations of the dual‐enzyme cascade reaction. g) Catalytic efficiency of GOx at varying mass ratios of GOx to CAT (*n* = 3). h) O_2_ content, i) H_2_O_2_ concentration, j) gluconic acid concentration, and k) pH levels in different reaction groups (*n* = 3). **p < 0.01, ***p < 0.001, and ****p < 0.0001. Statistical significance was calculated via the two‐tailed Student's *t*‐test.

An efficient proton generator (GC@SiO_2_) was synthesized by in situ encapsulating GOx and CAT within mesoporous silica nanocapsules using an interfacial confined sol‐gel process in an inverse (water‐in‐oil) miniemulsion (Figure 2a).^[^ ^28^, ^29^ ^]^ The enzymes were dissolved in an aqueous phase and emulsified to form nanodroplets dispersed in a cyclohexane continuous phase. Silica precursors were then introduced to form GC@SiO_2_ with a liquid core and a semipermeable shell. This process enabled the in situ encapsulation of enzymes in the interior of silica nanocapsules during their formation. The large internal cavity of capsule serves for cargo storage, while the mesoporous silica shell provides protection to loaded enzymes and facilitates selective permeation of low molecular weight substrates. The resulting GC@SiO_2_ was then transferred to an aqueous medium containing a PEG‐based surfactant (Lutensol AT50) to ensure stability in biological environments during subsequent applications. All the SiO_2_‐based nanocapsules, including blank SiO_2_, G@SiO_2_ (encapsulating GOx), C@SiO_2_ (encapsulating CAT), and GC@SiO_2_ (encapsulating both GOx and CAT), exhibited consistent nanocapsule‐like morphology with a defined core‐shell structure (Figures 2b and S1a), confirming that enzyme encapsulation did not alter the structural integrity of nanocapsules. Dynamic light scattering (DLS) analysis revealed that all nanocapsules displayed a uniform diameter of approximately 200 nm with a good dispersibility (Figures 2b and S1b), consistent with dimensions observed via transmission electron microscopy (TEM). Furthermore, zeta potential of the nanocapsules remained unchanged at approximately −20 mV after enzyme encapsulation (Figure S2).

To assess the encapsulation efficiency of enzymes in GC@SiO_2_, the samples were repeatedly centrifuged to separate the GC@SiO_2_ from nonencapsulated free enzymes. Pellets from each centrifugation step (P_1_–P_6_) and the final supernatant (SN_6_) were collected, and enzyme contents were determined using a BCA assay. For GC@SiO_2_, an encapsulation efficiency exceeding 97% was achieved (Figures 2c and S3). To verify the co‐encapsulation of both enzymes within mesoporous silica nanocapsules, high‐resolution confocal laser scanning microscopy (CLSM) was used to investigate the co‐localization of FITC‐labeled SiO_2_ nanocapsules (SiO_2_‐FITC), AF405‐labeled GOx (GOx‐AF405), and AF555‐labeled CAT (CAT‐AF555) (Figures 2d and S4). CLSM images confirmed a high degree of enzyme co‐localization within the nanocapsules. Quantitative co‐encapsulation of GOx and CAT was further validated by comparing the fluorescence intensity (FI) ratios of GOx‐AF405 and CAT‐AF555 before and after encapsulation in GC@SiO_2_ (Figure 2e). The FI(GOx):FI(CAT) ratio in free solution was 0.75 ± 0.02, closely matching that in GC@SiO_2_ (0.80 ± 0.01), demonstrating a precise and controllable encapsulation of enzyme combinations based on stoichiometric input. Moreover, GC@SiO_2_ maintained consistent hydrodynamic sizes and polydispersity indexes over 1 month at 4 °C (Figure S5a–c), and both enzymes retained ∼95% of their initial activity after 2 weeks of storage at 4 °C (Figure S5d,e), confirming the excellent storage stability. These results highlight the robust encapsulation performance and long‐term stability of GC@SiO_2_ as a proton generator.

Glucose oxidation catalyzed by GOx produces gluconic acid, which lowers the pH within the GC@SiO_2_ cavity (I and III in Figure 2f). However, the intermediate byproduct H_2_O_2_ is cytotoxic and can impair the enzymatic activity of GOX. In the presence of CAT, this toxicity is alleviated by the decomposition of H_2_O_2_, while the generated O_2_ further promotes proton production by feeding the GOx‐mediated reaction (II and IV in Figure 2f). Consequently, optimizing the ratio of GOx to CAT is critical for maximizing the overall efficiency of this enzymatic cascade. To determine the optimal ratio, the relative activity of GOx was evaluated by systematically varying the GOx:CAT mass ratio. A combination of GOx and CAT demonstrated a significantly higher activity in glucose oxidation compared to GOx alone (Figure 2g), with the optimal GOx:CAT mass ratio determined to be 1:0.5. This ratio was used for subsequent GC@SiO_2_ synthesis.

The catalytic performance of GC@SiO_2_ was assessed by measuring concentrations of O_2_, H_2_O_2_, and gluconic acid, as well as pH changes across different groups. As shown in Figure 2h, no significant variation in O_2_ content was observed in the control group. However, the GC and GC@SiO_2_ groups exhibited higher O_2_ levels compared to the GOx and G@SiO_2_ groups, attributed to additional O_2_ generation by CAT. Notably, GC@SiO_2_ exhibited a higher O_2_ level than GC group, indicating an enhanced enzyme activity due to the confinement effect within the nanocapsule cavity.^[^ ^30^ ^]^ The confined space inside the GC@SiO_2_ cavity facilitated a high local concentration of H_2_O_2_, enabling its immediate utilization by co‐encapsulated CAT. In contrast, H_2_O_2_ in the free enzyme solution (GC group) was not consumed as efficiently, limiting the catalytic efficiency of GOx. The GC@SiO_2_ group displayed the lowest H_2_O_2_ concentration among all groups except the control (Figure 2i), confirming the efficient participation of CAT in the cascade reactions. Gluconic acid concentration was quantified using a specific colorimetric assay.^[^ ^31^ ^]^ In the presence of gluconic acid, hydroxylamine, and Fe^3+^, an orange‐red complex forms, exhibiting a characteristic absorption peak at 505 nm proportional to gluconic acid concentration (Figures S6 and S7). Among all groups, GC@SiO_2_ demonstrated the strongest absorption at 505 nm, indicating the highest gluconic acid production (Figures 2j and S8). To directly evaluate proton generation, pH changes were monitored during the enzymatic reaction. Compared with the control group, substantial pH decreases were observed in the G@SiO_2_ (from 7.4 to 5.4), GC (from 7.3 to 4.5), and GOx groups (from 7.5 to 5.2). The most pronounced decrease occurred in the GC@SiO_2_ group (from 7.4 to 3.8), underscoring its superior proton production capability (Figure 2k). Notably, the encapsulated enzymes retained their activity in the presence of protease K, highlighting the protective effect of the silica shell (Figure S9).

### Construction and Bioenergetic Evaluation of the Artificial Mitochondria

**Figure 3:**
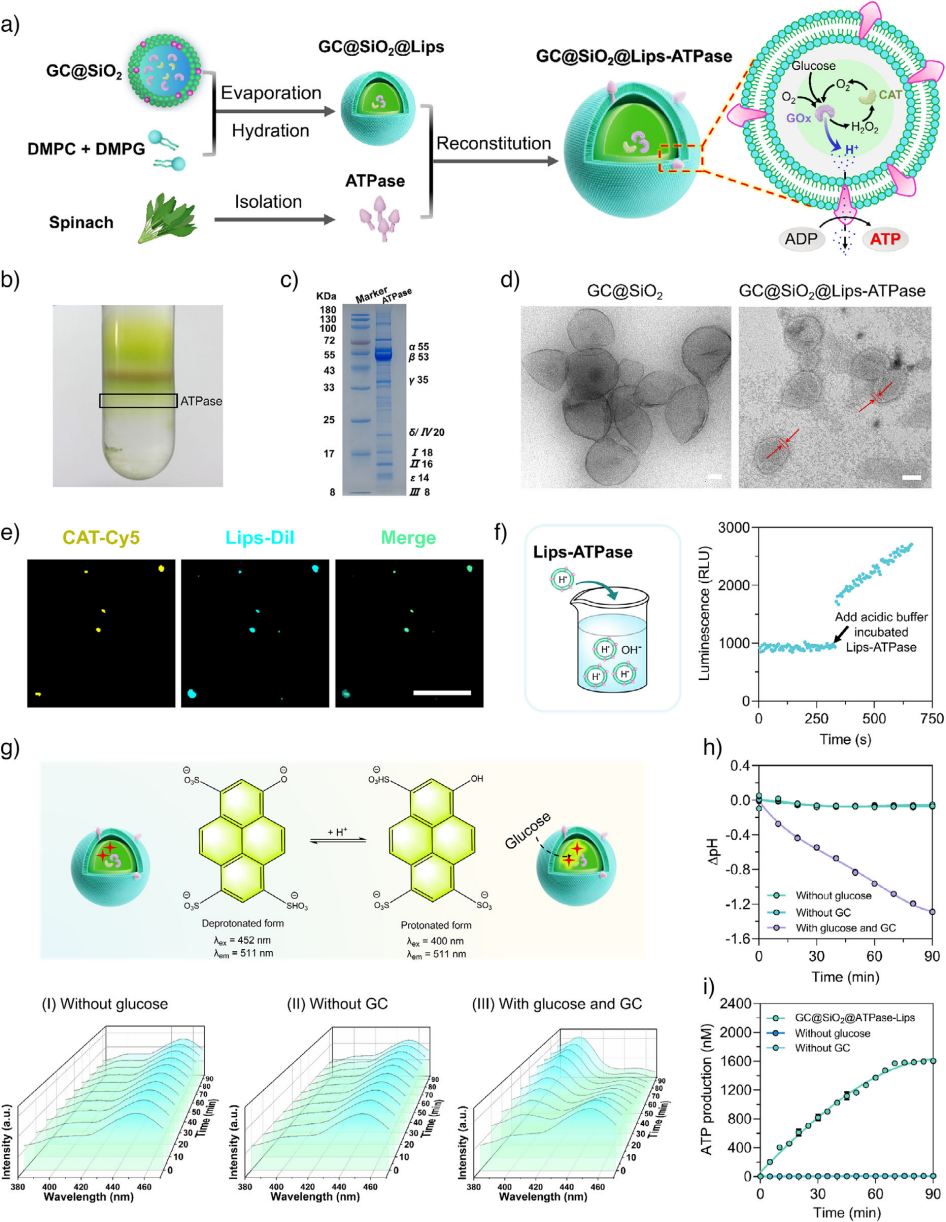
Construction of GC@SiO_2_@Lips‐ATPase and evaluation of its ATP generation capability. a) Schematic illustration of the sequential assembly of GC@SiO_2_@Lips‐ATPase. b) Photograph of purified ATPase obtained via sucrose density gradient centrifugation. c) SDS‐PAGE analysis of purified ATPase. d) TEM images of the GC@SiO_2_ and GC@SiO_2_@Lips‐ATPase. Scale bar: 50 nm. e) CLSM images showing the colocalization of fluorescently labeled components in GC@SiO_2_@Lips‐ATPase: Cy5‐labeled CAT (yellow) and DiI‐labeled liposomes (cyan). Scale bar: 10 µm. f) Fluorescence intensity change of basic buffer (pH 8.8) before and after adding Lips‐ATPase pre‐equilibrated in acidic buffer (pH 4.5). g) Top: Scheme illustrating the protonated and deprotonated forms of pyranine with corresponding excitation and emission wavelengths; Bottom: Time‐dependent fluorescence excitation spectra under different conditions: (I) without glucose, (II) without GC, and (III) with both glucose and GC. h and i) Time‐dependent pH changes and ATP production in various experimental groups.

Mitochondrial ATP synthesis, the primary cellular energy‐generating mechanism, relies on the spatial coupling of proton pumping and ATPase catalysis across biological membranes. To mimic this mechanism, we engineered a compartmentalized hybrid system (called GC@SiO_2_@Lips‐ATPase) by integrating two functional modules: 1) GC@SiO_2_ functions as a glucose‐driven proton pump via a GOx‐CAT enzymatic cascade, enabling efficient proton release, and 2) plant‐derived ATPase reconstituted into liposomes facilitates ATP synthesis by utilizing the proton gradient (Figure 3a).

ATPase was extracted from fresh spinach leaves via sucrose density gradient centrifugation (Figures 3b and S10). Sodium dodecyl sulfate‐polyacrylamide gel electrophoresis (SDS‐PAGE) analysis of the purified ATPase revealed the presence of both the hydrophobic F_0_ complex (I, II, III, and IV subunits) and the hydrophilic F_1_ complex (α, β, γ, δ, and ε subunits), confirming its structural integrity (Figure 3c). To construct GC@SiO_2_@Lips‐ATPase, GC@SiO_2_ was encapsulated within liposomes using a thin film hydration method, followed by detergent‐mediated ATPase reconstitution into the lipid bilayer.^[^ ^24^ ^]^ Specifically, DMPC and DMPG were dissolved in a methanol:chloroform mixture solvent, evaporated to form a lipid film, and subsequently hydrated with GC@SiO_2_ solution. The suspension was ultrasonicated and filtered through a 0.22 µm membrane to obtain GC@SiO_2_@Lips. The empty liposome in GC@SiO_2_@Lips‐ATPase was removed by centrifugation, ensuring >97% purity of GC@SiO_2_@Lips for subsequent ATPase integration (Figure S11). For ATPase integration, GC@SiO_2_@Lips were incubated with Triton X‐100‐solubilized ATPase, followed by detergent removal using Bio‐beads SM‐2 to facilitate ATPase integration. SDS‐PAGE and circular dichroism (CD) analysis confirmed successful ATPase reconstitution, as Lips‐ATPase exhibited identical electrophoretic profiles to pure ATPase (Figure S12). CD spectra revealed the retention of its native α‐helix structure, with characteristic double negative peaks at 210 and 225 nm (Figure S13). Collectively, these results verified structural preservation and functional integration of ATPase into the lipid bilayer.

TEM imaging revealed the well‐defined core‐shell architecture of GC@SiO_2_@Lips‐ATPase, in which the silica nanocapsule (GC@SiO_2_) was enveloped by a continuous lipid bilayer membrane (Figures 3d and S14). The measured membrane thickness of 5–6 nm matches that of a phospholipid bilayer,^[^ ^32^ ^]^ confirming successful liposome coating. Fluorescence imaging demonstrated a strong co‐localization of Cy5‐labeled CAT (CAT‐Cy5) within GC@SiO_2_ and DiI‐labeled liposomes (Lips‐DiI), validating the spatial integration of the functional modules (Figure 3e). DLS analysis showed an increase in particle size from 199 ± 4 nm (GC@SiO_2_) to 233 ± 8 nm (GC@SiO_2_@Lips‐ATPase) with minimal zeta potential variation (Figure S15). Encapsulation efficiency of GC@SiO_2_ in liposomes was about 90% according to the CLSM results (Figure S16). Besides, an average of ∼16.4 ATPase molecules were integrated per GC@SiO_2_@Lips‐ATPase. The proton‐translocating activity of reconstituted ATPase was examined using a pH‐jump‐driven ATP synthesis assay (Figure 3f). When the Lips‐ATPase, pre‐equilibrated in an acidic buffer (pH 4.5), was exposed to a basic buffer (pH 8.8), the ATP signal was rapidly generated, demonstrating its ability to harness proton gradients for ATP synthesis.

**Figure 4:**
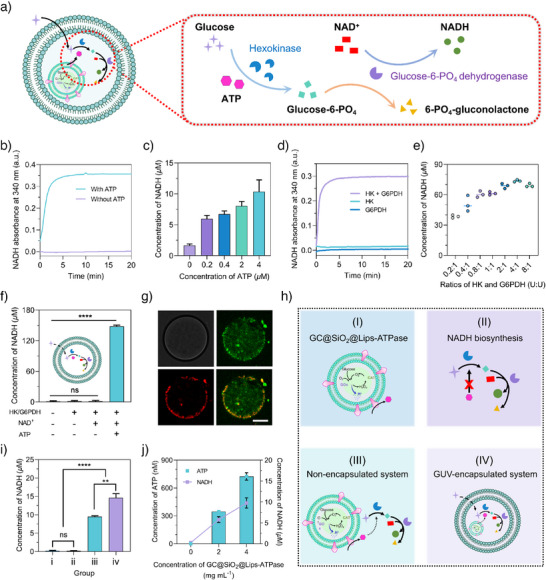
ATP self‐supply for NADH biosynthesis in artificial cells. a) Schematic illustration of the enzymatic cascade for ATP‐dependent NADH biosynthesis from glucose. b) Time‐dependent NADH production in bulk solution with or without ATP. c) NADH production at varying ATP concentrations after 20 min of reaction. d) NADH production in the presence of a single enzyme (HK or G6PDH) or dual enzymes (HK and G6PDH). e) NADH production at different HK/G6PDH ratios. f) NADH yields across different treatment groups at 30 min: Control group; with HK/G6PDH; with HK/G6PDH and NAD^+^; with HK/G6PDH, NAD^+^, and ATP. g) CLSM images showing GC@SiO_2_@Lips‐ATPase in GUVs. FITC‐labeled SiO_2_@Lips‐ATPase (green) and DiI‐labeled GUVs (red). Scale bar: 10 µm. h) Schematic diagram of the four reaction groups: I) GC@SiO_2_@Lips‐ATPase alone (without NADH biosynthesis pathway); II) NADH biosynthesis pathway alone (without ATP production module); III) nonencapsulated GC@SiO_2_@Lips‐ATPase and NADH biosynthesis pathway; IV) GUV‐encapsulated system of GC@SiO_2_@Lips‐ATPase and NADH biosynthesis pathway. i) NADH yields of the four groups at 90 min. j) NADH and ATP production at different GC@SiO_2_@Lips‐ATPase concentrations in GUVs at 90 min. ns represented no statistical difference, **p < 0.01, ***p < 0.001, and ****p < 0.0001. Statistical significance was calculated via the two‐tailed Student's *t*‐test.

To evaluate the bioenergetic performance of GC@SiO_2_@Lips‐ATPase, proton flux dynamics were tracked using pyranine, a ratiometric pH‐sensitive fluorophore, based on its excitation wavelength‐dependent fluorescence shift (F_452_/F_400_ ratio). As shown in Figure 3gI–III, control groups lacking either glucose (I) or GC module (II) exhibited minimum pH variations. Notably, when both glucose and GC were present (III), a progressive increase in fluorescence at 400 nm (Δ*F* ≈ 76.2%) and a corresponding decrease in F_452_/F_400_ ratio (Δ*R* ≈ −83.7%) were observed over 90 min (Figure S17), confirming glucose‐driven proton accumulation. Third‐order polynomial regression analysis of ratiometric data (Figure S18) quantified an intrasystem acidification (ΔpH) of 1.3 ± 0.2 units (Figure 4h), surpassing the ΔpH threshold (1.0) necessary for native ATPase activation.^[^ ^33^ ^]^

ATP synthesis capacity of GC@SiO_2_@Lips‐ATPase was quantitatively assessed through kinetic profiling. ATP assays revealed sustained ATP accumulation, reaching a maximum yield of 1.6 µM at a rate of 20–40 nmol min^−1^ (Figures 3i and S19,S20). This efficient ATP production was driven by a stable proton motive force (ΔpH ≈ 1.4), established through the synergistic coupling of the GC@SiO_2_ cascade (glucose oxidation‐driven proton pumping) and ATPase phosphorylation. To systematically examine the effect of glucose concentration on the GC@SiO_2_@Lips‐ATPase system, both the transmembrane proton gradient and ATP production across a range of glucose concentrations (1.5–50 mM) were quantified. As shown in Figure S21, both the transmembrane proton gradient and ATP production exhibited a strong positive correlation with increasing glucose concentration, providing a clearer assessment of the catalytic efficiency and energy transduction performance of GC@SiO_2_@Lips‐ATPase. Furthermore, recognizing that glucose‐to‐ATP conversion efficiency is a key performance metric, we determined this by correlating glucose feeding concentrations with ATP yields over the enzymatic cascade. As shown in Figure S22, the efficiency increased at lower glucose concentrations (<5 mM) but decreased at higher concentrations (>5 mM), with a maximum value exceeding 600 nmol ATP per µmol glucose. To verify whether GC@SiO_2_@Lips‐ATPase refuels with additional glucose, we measured ATP production upon sequential glucose supplementation. As shown in Figure S23, the second glucose supplementation moderately enhanced ATP output, yielding an approximate 25% increase. These results confirm the efficient integration between the proton pumping module and ATPase catalytic activity, mirroring natural mitochondrial energy transduction.

### NADH Biosynthesis Powered by Artificial Mitochondria in Artificial Cells

NADH, a central electron carrier in mitochondrial oxidative phosphorylation, regulates the cellular energy flux through NAD^+^/NADH redox cycling.^[^ ^34^, ^35^, ^36^ ^]^ To assess the energy‐generating capacity of GC@SiO_2_@Lips‐ATPase as an artificial mitochondrial mimic, an ATP‐driving NADH biosynthesis pathway was established in artificial cells (Figure 4a). In this cascade, hexokinase (HK) catalyzes glucose phosphorylation in the presence of ATP, producing glucose‐6‐PO_4_ (G6P), which is subsequently oxidized by glucose‐6‐PO_4_ dehydrogenase (G6PDH) to 6‐PO_4_‐gluconolactone, concurrently reducing NAD^+^ to NADH.

The ATP‐driving conversion of NAD^+^ to NADH was assessed in bulk solution, where NADH exhibited a characteristic absorption peak at 340 nm (Figure S24). Compared to ATP‐free conditions, the addition of ATP significantly enhanced NADH absorption at 340 nm in a concentration‐dependent manner (Figure 4b,c), confirming ATP's indispensable role. Optimization studies revealed that NADH biosynthesis was critically dependent on substrate concentrations and enzyme stoichiometry. NADH yield reached a plateau at 400 µM glucose and 160 µM NAD^+^ (Figure S25a,b), indicating a limitation imposed by enzyme kinetics. Notably, an optimal HK:G6PDH activity ratio of 4:1 balances ATP consumption (HK‐driven phosphorylation) with NAD^+^ reduction (G6PDH‐mediated oxidation), maximizing catalytic efficiency (Figure 4d,e). These results established key operational parameters for implementing ATP‐driving NADH biosynthesis in artificial cells.

To mimic cellular compartmentalization, we encapsulated the NADH biosynthesis pathway within giant unilamellar vesicles (GUVs) derived via droplet‐transfer, creating a spatially organized reaction system (Figure 4f). Approximately 85% of G6PDH and hexokinase were encapsulated in GUVs, demonstrating the superior encapsulation capacity of the droplet transfer method. ATP‐loaded artificial cells exhibited significantly higher NADH production than ATP‐deficient controls, validating GUVs as effective platforms for orchestrating energy‐coupled metabolic pathways.

To further integrate ATP synthesis with NADH biosynthesis, GC@SiO_2_@Lips‐ATPase was co‐encapsulated with the NADH biosynthesis pathway in GUVs. Specifically, an inner aqueous phase containing NAD^+^, HK, G6PDH, and GC@SiO_2_@Lips‐ATPase was first emulsified in a paraffin oil solution containing lipids (DMPC and cholesterol) to form water‐in‐oil (W/O) emulsion. The emulsion was then layered on top of an outer aqueous phase (300 mM galactose), and GUVs were harvested from the bottom layer of the tube after centrifugation. CLSM imaging confirmed the successful preparation of GUVs (Figure S26) and also the encapsulation of FITC‐labeled SiO_2_@Lips‐ATPase (green) within DiI‐labeled GUVs (red, Figure 4g). Functional coupling between the ATP synthesis and NADH production pathways was assessed through four experimental groups (Figure 4h): I) GC@SiO_2_@Lips‐ATPase alone (without NADH biosynthesis pathway); II) NADH biosynthesis pathway alone (without ATP); III) nonencapsulated GC@SiO_2_@Lips‐ATPase and NADH biosynthesis pathway; IV) GUV‐encapsulated system of GC@SiO_2_@Lips‐ATPase and NADH biosynthesis pathway. Quantitative analysis showed negligible NADH production in groups I and II, while group IV exhibited a 53.6% increase compared to group III (Figure 4i). These results demonstrated that compartmentalization by GUV enhances the catalytic efficiency of ATP‐coupled enzyme cascades (III versus IV) and confirmed enzyme‐ and ATP‐dependency in NADH biosynthesis (I and II versus III and IV). Furthermore, NADH yield in artificial cells increased by approximately 69% as ATP concentration generated by GC@SiO_2_@Lips‐ATPase rose from 347 nM to 725 nM (Figure 4j).

Collectively, these findings demonstrate that GUV‐encapsulated GC@SiO_2_@Lips‐ATPase mimics mitochondrial energy coupling by converting glucose‐driven proton gradients (via GC@SiO_2_) into ATP synthesis, which fuels compartmentalized NADH production through an ATP‐dependent enzyme cascade (HK and G6PDH). By integrating bioenergetic modules within cell mimics, this system achieves the NAD^+^/NADH redox coupling central to mitochondrial oxidative phosphorylation.

## Conclusions

In summary, we developed an efficient mitochondria‐mimicking ATP generator (GC@SiO_2_@Lips‐ATPase) to enable autonomous energy supply and metabolic coupling in artificial cells. This ATP generator consists of an inner mesoporous silica nanocapsule co‐loaded with GOX and CAT, encapsulated by an outer ATPase‐integrated liposome bilayer. In this system, GOx and CAT were quantitatively co‐encapsulated at their optimized ratio within mesoporous silica nanocapsules, where the silica shell enhanced enzyme stability and the confined internal space facilitated rapid decomposition of H_2_O_2_ into O_2_ via CAT catalysis. The generated O_2_ in turn fuels the GOx‐catalyzed reaction, resulting in sustained proton release and the establishment of a strong transmembrane proton gradient (pH shift from 7.37 to 3.77). This proton motive force effectively drove ATP synthase rotation, enabling high‐yield ATP production with a synthesis rate of 20–40 nmol min^−1^ and a maximum ATP yield of 1.6 µM, benefiting from enhanced stability and tight spatial coupling of enzymes. Furthermore, integration of the mitochondria‐mimicking ATP generator into artificial cells enabled glucose‐driven, continuous ATP generation, which powered ATP‐dependent NADH biosynthesis and successfully recapitulated oxidative phosphorylation. This work represents a significant step toward engineering energy‐autonomous artificial cells and provides a robust platform for investigating compartmentalized bioenergetics.

## Conflict of Interests

The authors declare no conflict of interest.

## Supporting information



Supporting Information

## Data Availability

The data that support the findings of this study are available from the corresponding author upon reasonable request.
