# Supplementary material for: Engineering Artificial Mitochondria with Self‐Amplifying Proton Generation for Autonomous Energy Supply and Metabolic Coupling in Artificial Cells
Source: Angew Chem Int Ed Engl. 2025 Sep 30;64(48):e202514980. doi: 10.1002/anie.202514980 (PMC12643332; doi:10.1002/anie.202514980)
Supplement: Supplementary file 1 — Supporting Information [file ANIE-64-e202514980-s001.docx]

Supporting Information

**Engineering Artificial Mitochondria with Self-Amplifying Proton Generation for Autonomous Energy Supply and Metabolic Coupling in Artificial Cells**

Fangqin Fu^+[a,b,c]^, Xuemei Hu^+[a,b,c]^, Shijia Tao^[a,b,c]^, Yu Gao^[a,b,c]^, Daniel Crespy^[d]^, Katharina Landfester^[e]^*, Xiangzhao Mao^[b,f]^*, and Shuai Jiang*^[a,b,c]^

[a] F. Fu, X. Hu, S. Tao, Y. Gao, Prof. S. Jiang

Key Laboratory of Marine Drugs, Chinese Ministry of Education, School of Medicine and Pharmacy

Ocean University of China

Qingdao 266003, China

E-mail: [jiangshuai@ouc.edu.cn](mailto:jiangshuai@ouc.edu.cn)

[b] F. Fu, X. Hu, S. Tao, Y. Gao, X. Mao, Prof. S. Jiang

State Key Laboratory of Marine Food Processing and Safety Control

Ocean University of China

Qingdao, 266404, PR China

[c] F. Fu, X. Hu, S. Tao, Y. Gao, Prof. S. Jiang

Laboratory for Marine Drugs and Bioproducts, Qingdao Marine Science and Technology Center

Qingdao 266237, China

[d] D. Crespy

Department of Materials Science and Engineering, School of Molecular Science and Engineering

Vidyasirimedhi Institute of Science and Technology (VISTEC)

Rayong 21210, Thailand

[e] K. Landfester

Max Planck Institute for Polymer Research

Ackermannweg 10, 55128 Mainz, Germany

E-mail: [landfester@mpip-mainz.mpg.de](mailto:landfester@mpip-mainz.mpg.de)

[f] X. Mao

College of Food Science and Engineering, Ocean University of China

Qingdao 266003, P. R. China

E-mail: [xzhmao@ouc.edu.cn](mailto:xzhmao@ouc.edu.cn)

[^+^] These authors contributed equally to this work.

Table of Contents

[Table of Contents 2](#_Toc196137890)

[1. Experimental Section 3](#_Toc196137891)

[1.1 Materials 3](#_Toc196137892)

[1.2 Synthesis of enzyme-loaded SiO_2_ nanocapsules 3](#_Toc196137893)

[1.3 Preparation of fluorescently labelled enzymes 3](#_Toc196137894)

[1.4 Isolation of chloroplast ATP synthase (ATPase) 3](#_Toc196137895)

[1.5 Preparation of GC@SiO_2_@Lips-ATPase 4](#_Toc196137896)

[1.6 Characterization of nanocapsules 4](#_Toc196137897)

[1.7 Preparation of GUVs containing GC@SiO_2_@Lips-ATPase 4](#_Toc196137898)

[1.8 Evaluation of enzyme encapsulation efficiency in GC@SiO_2_ 5](#_Toc196137899)

[1.9 Enzyme activity assessment of GC@SiO_2_ 5](#_Toc196137900)

[1.10 Identification of ATPase using sodium dodecyl sulfate-polyacrylamide gel electrophoresis (SDS-PAGE) 5](#_Toc196137901)

[1.11 Detection of ATPase activity in Lips-ATPase 5](#_Toc196137902)

[1.12 Determination of transmembrane proton gradient 5](#_Toc196137903)

[1.13 Detection of ATP production in GC@SiO_2_@Lips-ATPase 6](#_Toc196137904)

[1.14 Monitoring NADH output in bulk reactions 6](#_Toc196137905)

[Results 6](#_Toc196137906)

[References 14](#_Toc196137907)

1. Experimental Section

**1.1 Materials**

Catalase (CAT, ≥ 3000 U mg^-1^), tetramethoxysilane (TMOS, 98%), (3-aminopropyl) triethoxysilane (APTES, 99%), fluorescein 5(6)-isothiocyanate (FITC, 95%), glucose, 1,2-dimyristoyl-sn-glycero-3-phosphocholine (DMPC, > 99%), 1,2-dimyristoyl-sn-glycero-3-phospho-(1’-rac-glycerol) (sodium salt) (DMPG, > 99%), pyranine (> 98%), and paraffin oil were purchased from Aladdin Biochemical Technology Co., Ltd. Hydrogen peroxide (H2O2) content assay kit, 1,1-dioctadecyl-3,3,3,3- tetramethylindocarbocyanine iodide (DiI), oxidized nicotinamide adenine dinucleotide (NAD, > 98%), and reduced nicotinamide adenine dinucleotide (NADH, ≥ 98%) were obtained from Solarbio Science & Technology Co., Ltd. Glucose oxidase (GOx, > 180 U mg^-1^), polyglyceryl-6-polyricinoleate (PGPR, ≥ 75%), cyclohexane (≥ 99%), DL-1,4-dithiothreitol (DTT, 99%), Triton X-100 (10%), adenosine 5’-diphosphate disodium salt (ADP), and galactose (99%) were bought from Macklin Biochemical Technology Co., Ltd. SDS-PAGE precast gel, SDS-PAGE rapid electrophoresis buffer for Hepesv-Tris system, SDS-PAGE sample loading buffer (5X), and the Coomassie blue superfast staining solution were purchased from Yeasen Biotechnology Co., Ltd. Hexokinase (HK, 130 U mg^-1^) and glucose-6-PO4 dehydrogenase (G6PDH, 150 U mg^-1^) were obtained from Yuanye Biotechnology Co., Ltd. Bicinchoninic Acid (BCA, 96T) protein assay kit and enhanced ATP assay kit were purchased from Beyotime Biotechnology Co., Ltd. Bio-beads SM-2 were obtained from Bio-Rad Laboratories. All chemicals were used directly without further purification. Milli-Q water was used in all experiments.

**1.2 Synthesis of enzyme-loaded SiO_2_ nanocapsules**

SiO_2_ nanocapsules were synthesized using an inverse (water-in-oil) miniemulsion technique, adapted from a previously reported method.^[1, 2]^ The aqueous and oil phases were prepared separately. The aqueous phase contains a protein mixture (GOx:CAT = 2:1, wt/wt) pre-dissolved in PBS (10 mM, pH 7.4) at a concentration of 7.5 mg mL^-1^. The oil phase consisted of a cyclohexane solution of PGPR at 26.7 mg mL^-1^. For the preparation of the miniemulsion, 12 mL of the oil phase solution was added to 400 *µ*L of the aqueous phase under magnetic stirring at 1000 rpm. The mixture was stirred for 10 min for pre-emulsification. The emulsion was then subjected to ultrasonication (520 W power, 5 s on/5 s off) in an ice bath for 3 min, followed by the addition of a mixture of 134 *µ*L of TMOS and 40 *µ*L of APTES into the emulsion. The samples were kept stirring at 1000 rpm and room temperature for 12 h until the sol-gel reaction finished. To transfer the nanocapsules from the cyclohexane phase to an aqueous environment, 0.6 mL of nanocapsule dispersion was added dropwise into 6 mL of Lutensol AT50 solution (0.3 wt% in Milli-Q water) in ultrasonic bath over 5 min. Then the mixture was stirred in an unsealed bottle at 1000 rpm and room temperature for 24 h to completely evaporate the cyclohexane. The resulting nanocapsules were purified by centrifugation at 1760 g and 10 °C for 20 min. The precipitate of enzyme-loaded SiO_2_ nanocapsules was collected and re-dispersed in Milli-Q water. SiO_2_ nanocapsules encapsulating GOx, CAT, or GOx+CAT were designated as G@SiO_2_, C@SiO_2_, or GC@SiO_2_, respectively. To prepare fluorescently labelled SiO_2_ nanocapsules, 2 mL of FITC solution (1 mg mL^-1^ in methanol) was first reacted with APTES at a 1:10 molar ratio under stirring (500 rpm, room temperature) overnight to yield fluorescently labelled silica precursors (APTES-FITC). Subsequently, 20 *μ*L of the obtained APTES-FITC solution was combined with TMOS and incorporated into the miniemulsion for fluorescent nanocapsule synthesis.

**1.3 Synthesis of fluorescently labelled enzymes**

For the preparation of AF405-labelled GOx and AF555-labelled CAT, carbonate buffer saline (CBS, pH 8.7) was first prepared by dissolving 0.736 g of NaCl, 0.106 g of Na_2_CO_3_, and 0.756 g of NaHCO_3_ in 100 mL of Milli-Q water. Then, 128.8 *μ*L of AF405-NHS ester or 125.7 *μ*L of AF555-NHS ester (5 mg mL^-1^ in DMSO) was mixed with GOx or CAT solution (5 mg mL^-1^ in CBS buffer) at the molar ratio of 10:1 under stirring at 500 rpm and room temperature in the dark overnight. Finally, Milli-Q water was used as the dialysis medium and refreshed every 12 h to remove the unbound AF405 or AF555. After 36 h of dialysis, freeze-drying was performed to obtain powders of AF405-labelled GOx and AF555-labelled CAT.

**1.4 Isolation of chloroplast ATP synthase (ATPase)**

All buffer solutions used in this procedure were prepared according to Table a. ATPase was isolated and purified from fresh spinach through ultracentrifugation (Optima L-100XP, Beckman Coulter, Inc., USA).^[3]^

**Step 1:** 500 g of fresh spinach leaves were washed and homogenized in a pre-cooled blender in 500 mL of Buffer-1 to obtain solution 1.

**Step 2:** Solution 1 was immediately centrifuged at 500 g for 5 min, and the pellet containing starch and intact plant tissue was discarded to obtain solution 2.

**Step 3:** Solution 2 was further centrifuged at 11,000 g for 20 min to collect the pellet containing intact chloroplasts. The resulting pellet was resuspended in 200 mL of hypotonic Buffer-2 by pipetting. Then, the resuspension was incubated in an ice bath for 0.5 h to disrupt the intact chloroplasts by osmotic shock and to remove water-soluble stroma proteins from the outer surface of thylakoid membranes to obtain solution 3.

**Step 4:** Solution 3 was further centrifuged at 16,900 g for 0.5 h to collect thylakoid membrane as the pellet. The resulting pellet was resuspended in 200 mL of high-ionic strength washing Buffer-3 by pipetting and then stirred at 500 rpm for 15 min to wash thylakoid membranes to obtain solution 4.

**Step 5:** Solution 4 was centrifuged at 16,900 g for 25 min to collect washed thylakoid membrane as the pellet. Then, the pellet was resuspended in 5 mL of Buffer-4 to obtain solution 5.

**Step 6:** DTT was added into solution 5 at a final concentration of 50 mM under stirring at 300 rpm for 15 min. An equal volume of Buffer-5 was added to the above solution 5. After centrifugation at 208,000 g and 4 °C for 1 h, the supernatant was collected to obtain solution 6 containing thylakoid membrane proteins rich in ATPase.

**Step 7:** ATPase in solution 6 was precipitated using an ammonium sulfate cut from 30% to 45% saturation at pH 8.0. The solution was further centrifuged at 12,000 g for 10 min and then the pellet was resuspended in 3 mL of Buffer-6 to obtain solution 7 containing the crude enzyme solution of ATPase.

**Step 8:** The mixture of solution 7 and an equal volume of Buffer-7 were added on top of a sucrose density gradient solution with a mass fraction of 60%, 52%, 44%, 36%, 28%, and 20%. The sucrose gradient density centrifugation (242,000 g, 14 h) was performed to obtain the ATPase (in 44% layer). The obtained ATPase solution was stored in liquid nitrogen.

**Table a.** Composition of buffer solutions


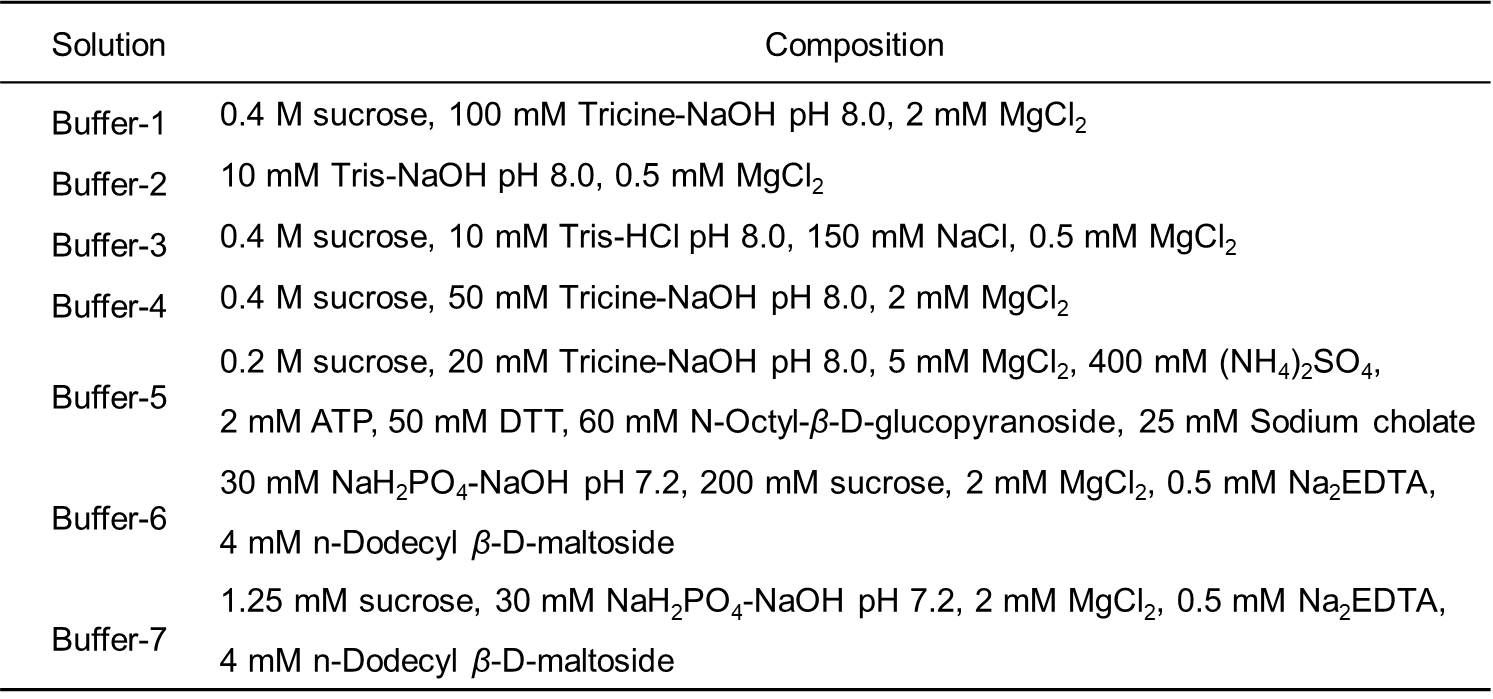


**1.5 Preparation of GC@SiO_2_@Lips-ATPase**

**Preparation of GC@SiO_2_@Lips.** GC@SiO_2_@Lips was prepared using a thin film hydration method.^[4]^ A lipid film was formed by dissolving 18 mg of DMPC and 2 mg of DMPG in 3 mL of mixture solvent of methanol:chloroform (1:1, v/v), followed by rotary evaporation (STRIKE 285, Ulab Technology, China) at 30 °C for 20 min. The lipid film was hydrated with 4 mL of GC@SiO_2_ solution under spinning at 37 °C for 20 min. The whole mixture was ultrasonicated (65 W, 1.5 s on/1.5 s off) in an ice bath for 15 min using an ultrasonic cell pulverizer (JY98-IIIDN, Ningbo Scientz Biotechnology Co., Ltd., China) and filtered through a 0.22-*μ*m membrane. The resulting solution was centrifuged at 1760 g for 20 min to collect the GC@SiO_2_@Lips as a pellet, which was re-dispersed in Milli-Q water. Blank-Lips and SiO_2_@Lips were prepared similarly without adding GC@SiO_2_ and GC, respectively. DiI-labelled GC@SiO_2_@Lips was prepared by mixing 30 *μ*L of DiI (1 mg mL^-1^) with the above mixture of DMPC and DMPG.

**Preparation of GC@SiO_2_@Lips-ATPase.** The obtained GC@SiO_2_@Lips (12 mg mL^-1^, 500 *μ*L), ATPase (80 *μ*L, 2.3 mg mL^-1^), reconstitution buffer (20 mM Tricine, 40 mM NaCl, and 5 mM MgCl_2_, 420 *μ*L), and Triton X-100 (10%, 80 *μ*L) were mixed and stirred at 500 rpm and 4 °C for 1 h. Then, 100 mg of Bio-beads SM-2 were added to adsorb Triton X-100 under stirring at 500 rpm for 1.5 h. Then the supernatant was transferred to a new tube. The addition and removal steps of Bio-beads SM-2 were repeated twice to obtain the GC@SiO_2_@Lips-ATPase. Lips-ATPase was prepared using the same method without GC@SiO_2_ addition.

**1.6 Characterization of nanocapsules**

Hydrodynamic diameter (*D*_h_) and Zeta potential of nanocapsules were measured at 25 °C using Malvern Zetasizer (Nano-ZS ZEN3600, Malvern Instruments Co., Ltd., UK). The morphology of nanocapsules was characterized using a transmission electron microscope (TEM, JEM-2100EX, JEOL Ltd., Japan).

**1.7 Preparation of GUVs containing GC@SiO_2_@Lips-ATPase**

GUVs were prepared using a droplet transfer method as previously reported.^[5]^ Specifically, a lipid solution (100 *μ*L, containing 4 mg mL^-1^ DMPC and 1 mg mL^-1^ cholesterol in chloroform) was added to 1 mL of paraffin oil. The mixture was heated at 80 °C for 1.5 h to create a lipid solution in paraffin. Afterwards, an inner phase solution (20 *μ*L of 300 mM sucrose solution containing 1 mM MgCl_2_,160 *μ*M NAD^+^, 4 U mL^-1^ HK, 1 U mL^-1^ G6PDH, and 4 *μ*M ATP or GC@SiO_2_@Lips-ATPase) was added into 200 *μ*L of the lipid solution in paraffin, followed by water bath sonication (ST5200-DT, Ningbo Scientz Biotechnology Co., Ltd., China) for 3 min. The emulsion was incubated in an ice bath for 10 min, and then added to the top of 200 *μ*L of pre-cooled outer phase solution (300 mM galactose) in a 1.5 mL plastic reaction tube and immediately centrifuged at 10,000 rpm and 4 °C for 0.5 h. Finally, GUVs were harvested by puncturing the bottom of the tube. DiI-labelled GUVs were prepared by mixing 30 *μ*L of DiI (1 mg mL^-1^) with the above lipid solution.

**1.8 Evaluation of enzyme encapsulation efficiency in** **GC@SiO_2_**

Encapsulation efficiency of enzymes in nanocapsules was determined using a centrifugation method. Briefly, 1 mL of GC@SiO_2_ dispersion was centrifuged at 1760 g and 10 °C for 20 min to separate GC@SiO_2_ from non-encapsulated free enzymes. The pellet was collected as P_1_, and the supernatant was transferred to a new tube for next centrifugation. This step was repeated for five times to obtain P_2_-P_6_. Enzyme contents in P_1_-P_6_ and the final supernatant (SN_6_) were determined using a BCA protein assay kit according to the manufacturer's instructions (Figure S3). The encapsulation efficiency of enzymes was calculated using the following equation:

EE (%) = (P_1_ + P_2_ + P_3_ + P_4_ + P_5_ + P_6_)/(SN_6_ + P_1_ + P_2_ + P_3_ + P_4_ + P_5_ + P_6_)*100%

where P_n_ represents the pellet collected from centrifugation step (n), and SN_6_ represents the supernatant obtained from the final centrifugation step.

**1.9 Enzyme activity assessment of GC@SiO_2_**

Oxygen content, gluconic acid concentration, pH change, and H_2_O_2_ concentration were determined for assessing the enzyme activity of GC@SiO_2_.

**Determination of oxygen content**. 5 mL of glucose solution (50 mM) was added to 50 *μ*L of free GOx solution (5 mg mL^-1^), mixture of free GOx and CAT (GC, 7.5 mg mL^-1^), G@SiO_2_ solution (5 mg mL^-1^), or GC@SiO_2_ solution (7.5 mg mL^-1^). Real-time change of oxygen content in different solutions was monitored using a dissolved oxygen meter (JPBJ-608, INESA Scientific Instrument Co., Ltd., China).

**Determination of gluconic acid concentration.** 1 mL of glucose solution (50 mM) was incubated with 200 *μ*L of GOx solution (5 mg mL^-1^), GC solution (7.5 mg mL^-1^), G@SiO_2_ solution (5 mg mL^-1^), or GC@SiO_2_ solution (7.5 mg mL^-1^) for 1 h. After centrifugation at 1760 g for 10 min, the supernatant was collected and mixed with 500 *μ*L of solution 1 (5.54 mM EDTA and 0.15 mM Et_3_N) and 50 *μ*L of solution 2 (6.68 M NH_2_OH), further incubating for 25 min. Subsequently, 250 *μ*L of solution 3 (2.69 M HCl, 0.1 M FeCl_3_, and 0.25 mM CCl_3_COOH) was added into the above mixture to obtain the red hydroxylamate-Fe^3+^ complex with absorbance at 505 nm.^[6]^ Finally, the absorbance of solutions at 505 nm was measured to evaluate the concentration of gluconic acid.

**Determination of pH variation.** 2 mL of glucose solution (50 mM) was incubated with 500 *μ*L of GOx solution (5 mg mL^-1^), GC solution (7.5 mg mL^-1^), G@SiO_2_ solution (5 mg mL^-1^), or GC@SiO_2_ (7.5 mg mL^-1^). The real-time pH values in different solutions were monitored using a pH meter (FE28-standard, Mettler-Toledo International Inc., China).

**Detection of** **H_2_O_2_ concentration.** 5 mL of glucose solution (50 mM) was incubated with 100 *μ*L solution of GOx (5 mg mL^-1^), GC (7.5 mg mL^-1^), G@SiO_2_ (5 mg mL^-1^), or GC@SiO_2_ (7.5 mg mL^-1^) for 1 h. The H_2_O_2_ concentration was measured using an H_2_O_2_ assay kit according to the manufacturer's instructions.

**1.10 Identification of ATPase using sodium dodecyl sulfate-polyacrylamide gel electrophoresis (SDS-PAGE)**

The purification of ATPase and its reconstitution in Lips-ATPase were confirmed using SDS-PAGE analysis on a 12% polyacrylamide gel. A mixture of bromophenol blue SDS loading buffer and solution of extracted ATPase (or Lips-ATPase) at a 1:4 volume ratio was heated at 100 °C for 5 min. After cooling to room temperature, SDS-PAGE analysis was conducted at a voltage of 150 V for 45 min. The gel was stained overnight with coomassie brilliant blue G-250 solution and subsequently rinsed with Milli-Q water three times to visualize the electrophoretic bands.

**1.11 Activity detection of ATPase in Lips-ATPase**

The enzymatic activity of ATPase reconstituted into liposomes was assessed using a luciferase assay.^[7]^

**Initial reaction mixture preparation.** Briefly, 20 *μ*L of 200 mM DTT solution was added to Lips-ATPase (6 mg mL^-1^) at a 1:4 volume ratio. The mixture was then incubated with 100 *μ*L of acidic buffer (pH 4.5, 20 mM succinate buffer, 2.5 mM MgCl_2_, and 10 mM NaH_2_PO_4_) for 0.5 h to activate ATPase.

**Baseline luminescence measurement (basic buffer control).** Subsequently, 100 *μ*L of basic buffer (pH 8.8, 200 mM Tricine, 0.2 mM ADP, 2.5 mM MgCl_2_, and 10 mM NaH_2_PO_4_) was added to each well, following by adding 50 *μ*L of luciferin-luciferase detection reagent mixture. Luminescence intensity (baseline signal, I_0_) was immediately measured using a microplate reader (Tecan Spark, Tecan, Switzerland).

**Acid-treated sample addition and luminescence measurement.** After baseline measurement, 100 *μ*L of the acidic buffer-incubated DTT/Lips-ATPase mixture was introduced into the pre-measured wells containing the basic buffer and detection reagents. Luminescence intensity was measured again (activated signal, I_1_).

**ATPase activity analysis.** ATPase activity was determined by calculating luminescence intensity difference before and after adding the acidic buffer-incubated Lips-ATPase, using the following equation: ΔI = I_1_ - I_0_.

**1.12 Determination of transmembrane proton gradient**

The transmembrane proton gradient generated by glucose oxidation was determined by monitoring the changes in the excitation spectra of the pH-sensitive fluorescent probe (pyranine) under acidic and basic conditions.^[8]^ The ratio of F_452_/F_400_ in the excitation spectra of pyranine correlates directly with proton concentration. The transmembrane proton gradient was determined by establishing a standard curve of pH versus F_452_/F_400_ and deriving a corresponding equation.

To establish the standard curve, 1 mL of PBS (0.01 M) at varying pH levels (pH 4.5, 5, 5.5, 6, 6.5, 7, 7.4, or 8.5) was mixed with 1 *μ*L of pyranine (1 *μ*M) in a 1.5 mL Eppendorf tube. The excitation spectra of the resulting solutions were scanned from 380 to 470 nm at an emission wavelength of 511 nm using a microplate reader (Tecan Spark, Tecan, Switzerland). The F_452_/F_400_ ratios were calculated for each pH level to generate the standard curve of pH versus F_452_/F_400_. Based on the standard curve, a third-order equation was derived from this curve, enabling pH determination.

To determine the transmembrane proton gradient of GC@SiO_2_@Lips-ATPase, 400 *μ*L of GC solution containing pyranine (2 mM) was used as the aqueous phase to prepare SiO_2_, which was further used to construct the GC@SiO_2_@Lips-ATPase. 500 *μ*L of GC@SiO_2_@Lips-ATPase (6 mg mL^-1^) containing pyranine was added to 2 mL of glucose solution (50 mM), and the excitation spectra of the solution were scanned from 380 nm to 470 nm at an emission wavelength of 511 nm using a microplate reader (Tecan Spark, Tecan, Switzerland).

**1.13 Detection of ATP production in GC@SiO_2_@Lips-ATPase**

ATP production was measured using a luminescence-based assay. For enzyme reduction, 20 *μ*L of DTT (200 mM) was added to 60 *μ*L of GC@SiO_2_@Lips-ATPase (6 mg mL^-1^). The resulting mixture was then combined with 100 *μ*L of basic buffer (pH 8.8, 200 mM Tricine, 0.2 mM ADP, 2.5 mM MgCl_2_, and 10 mM NaH_2_PO_4_). Following an incubation for 0.5 h, 100 *μ*L of glucose solution (50 mM) was added. ATP production over time was monitored using an ATP assay kit according to the manufacturer's instructions. The systems in the absence of glucose, GC, or ATPase were used as controls.

**1.14 Monitoring NADH output in bulk reactions**

A reaction mixture containing 1 mL of glucose (50 mM), 250 *μ*L of NAD^+^ (160 *μ*M), 50 *μ*L of MgCl_2_ (1 mM), 20 *μ*L of HK (4 U mL^-1^), 20 *μ*L of G6PDH (1 U mL^-1^), and 100 *μ*L of ATP (2 *μ*M) was prepared. The absorbance of NADH at 340 nm was monitored for 20 min using a microplate reader (Tecan Spark, Tecan, Switzerland). NADH concentration was calculated based on a standard curve (Figure S17).

Results


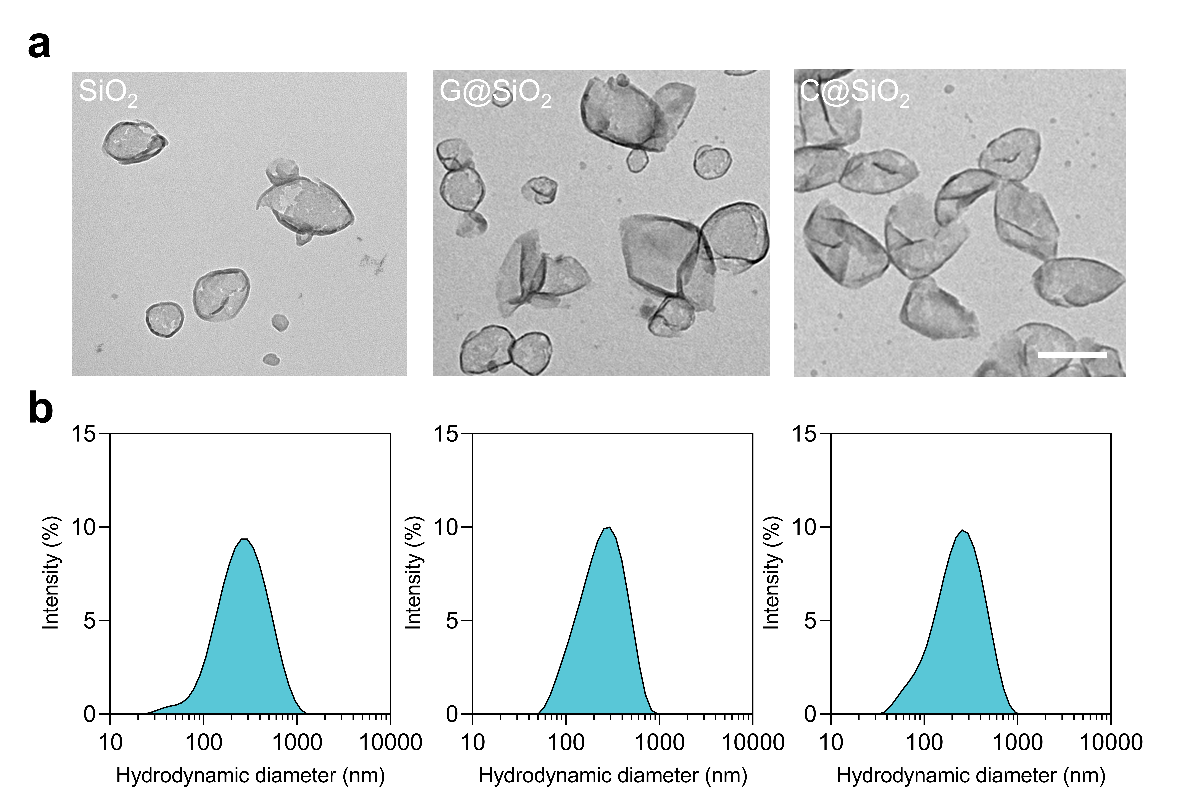


**Figure S1.** (a) TEM images of SiO_2_ nanocapsules, G@SiO_2_, and C@SiO_2_. Scale bar: 200 nm. (b) Size distribution of SiO_2_ nanocapsules, G@SiO_2_, and C@SiO_2_.


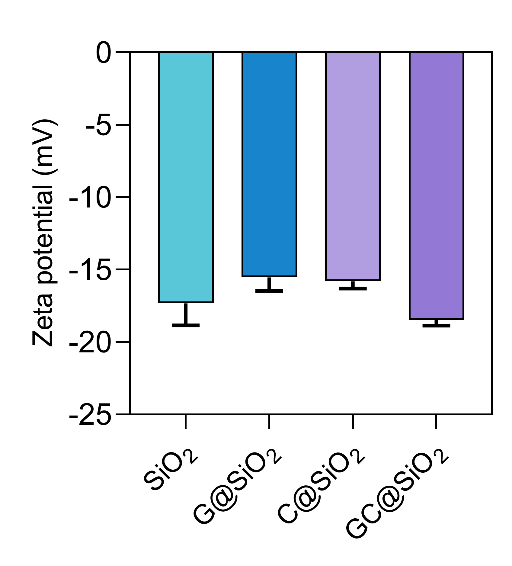


**Figure S2.** Zeta potential of SiO_2_ nanocapsules, G@SiO_2_, C@SiO_2_, and GC@SiO_2_ (n = 3).


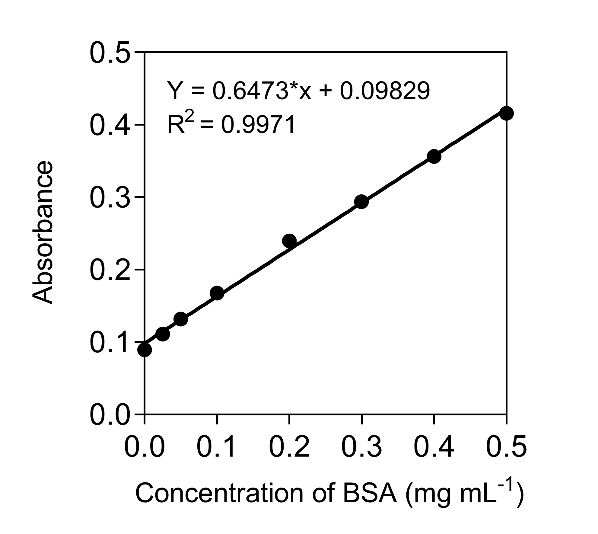


**Figure S3.** Quantitative standard curve of bovine serum albumin (BSA). BSA was used as the standard substance, and the concentration range was 0-0.5 mg mL^-1^.


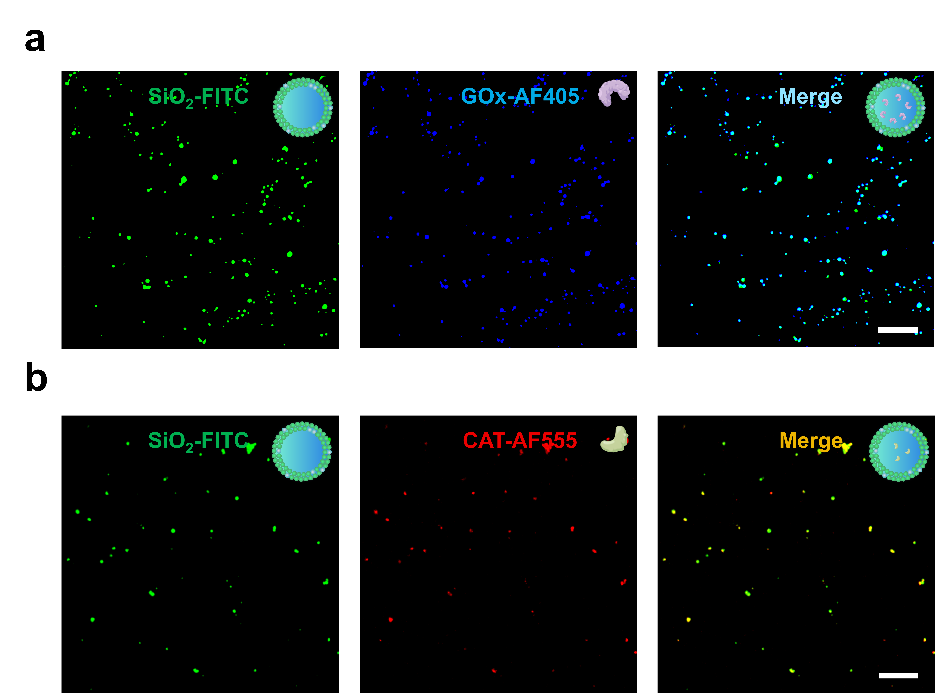


**Figure S4.** CLSM images of (a) G@SiO_2_ and (b) C@SiO_2_. SiO_2_ nanocapsules were labelled with FITC (green), GOx was labelled with AF405 (blue), and CAT was labelled with AF555 (red). Scale bar: 5 *μ*m.


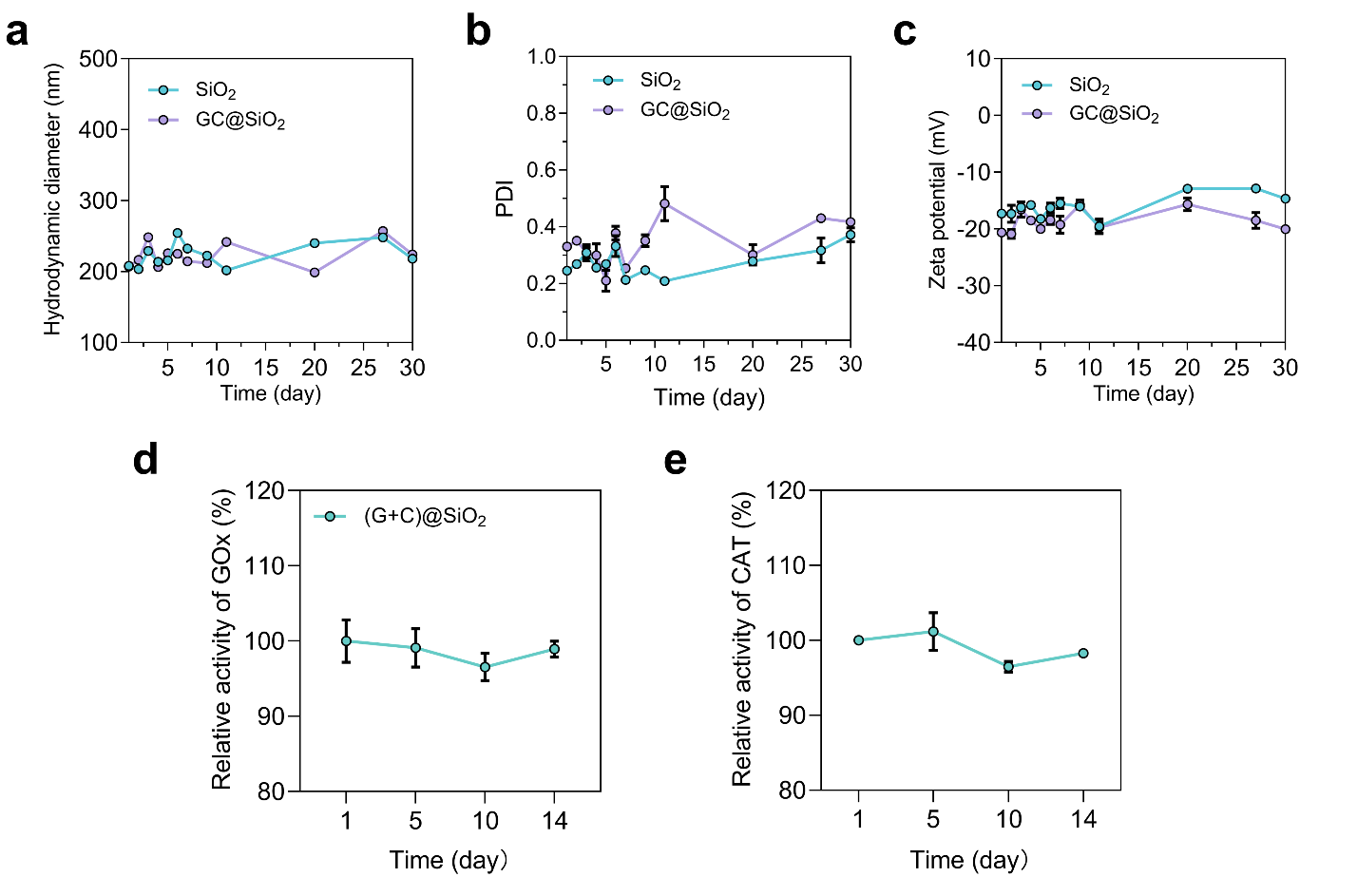


**Figure S5.** (a) Hydrodynamic diameters (*D*_h_), (b) polydispersity index (PDI), and (c) zeta potential of SiO_2_ nanocapsules and GC@SiO_2_ over a 30-day period (n = 3).


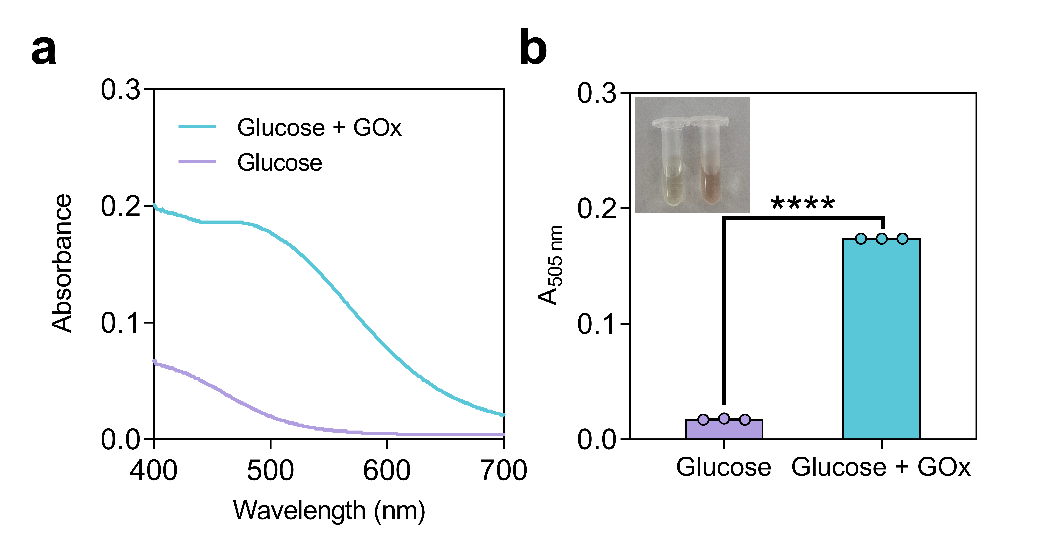


**Figure S6.** Determination of gluconic acid concentration. (a) Ultraviolet-visible (UV-Vis) spectra of the orange-red complex in glucose solutions with and without GOx addition. (b) Absorbance of gluconic acid at 505 nm and the corresponding color change of the solutions (n = 3). ****p < 0.0001. Statistical significance was calculated via the two-tailed Student's *t*-test.


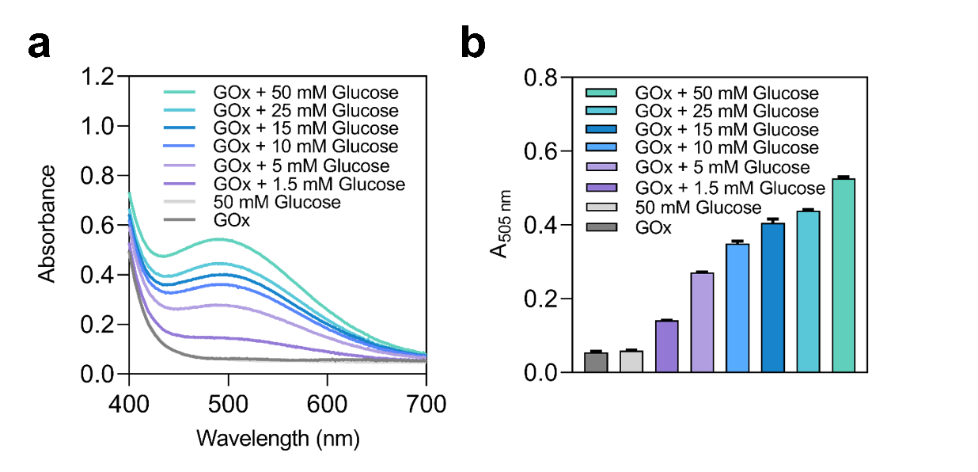


**Figure S7.** Determination of gluconic acid concentration. (a) UV-Vis spectra of the orange-red complex in enzymatic reaction groups with varying glucose concentrations. (b) Corresponding absorbance of gluconic acid at 505 nm (n = 3).


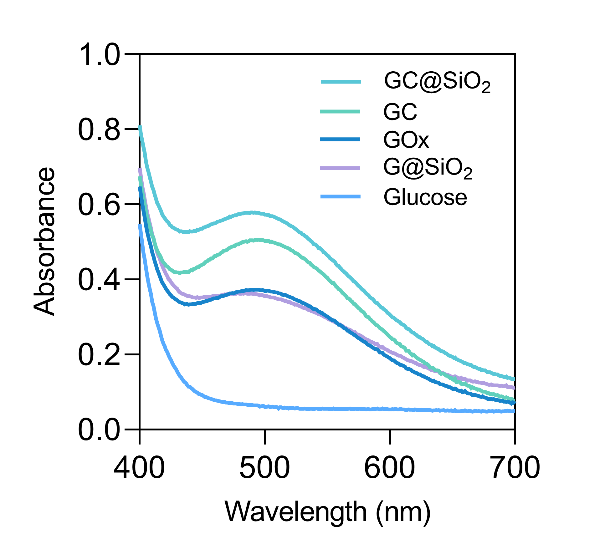


**Figure S8.** UV-Vis spectra of the orange-red complex in different enzymatic reaction groups.


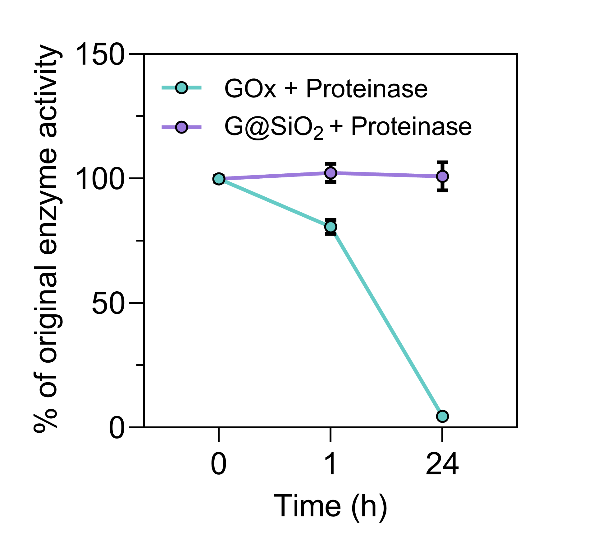


**Figure S9.** Remaining activity of GOx after incubation with protease K for 0 h, 1 h, and 24 h before and after encapsulation in SiO_2_ nanocapsules (n = 3).


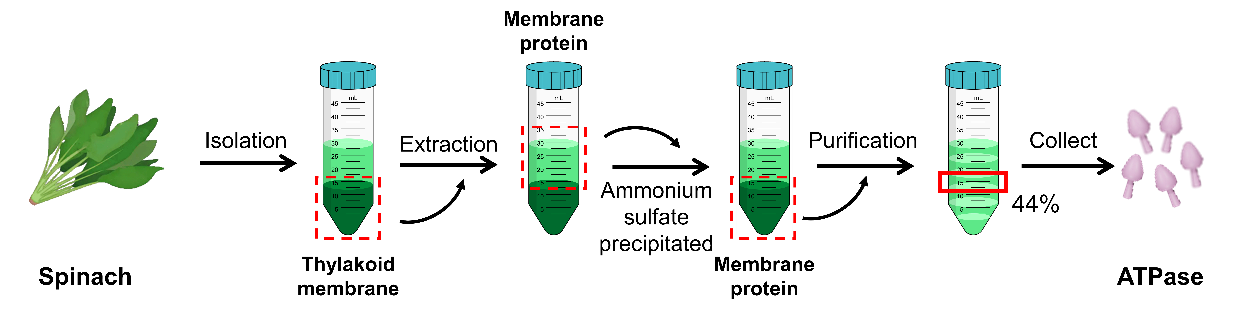


**Figure S10.** Schematic illustration of the extraction procedure of ATPase from spinach leaves through sucrose density gradient centrifugation.


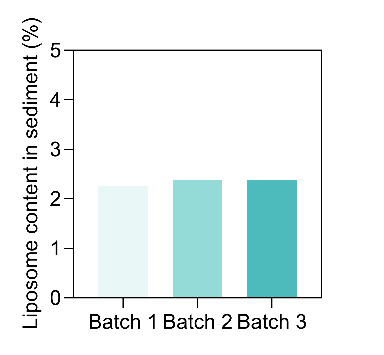


**Figure S11.** Liposome content in the sediment after centrifugation in three independent batches.


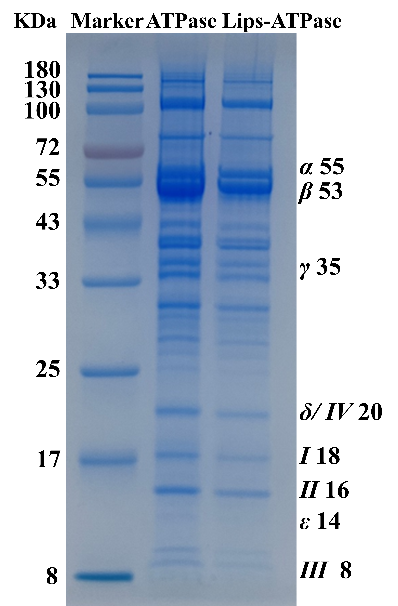


**Figure S12.** SDS-PAGE analysis of ATPase before and after reconstitution into liposomes (Lips-ATPase).


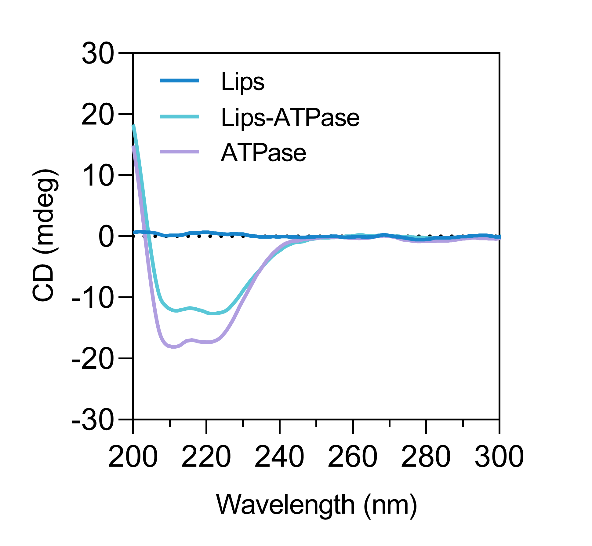


**Figure S13.** CD spectra of Lips, ATPase, and Lips-ATPase.


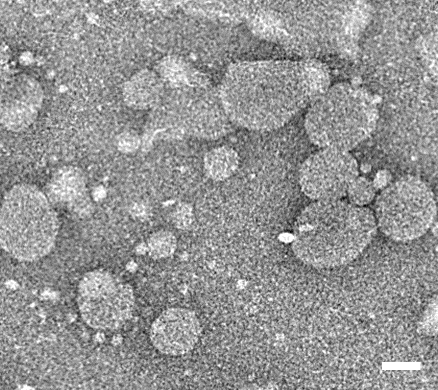


**Figure S14.** TEM image of liposomes. Scale bar: 50 nm.


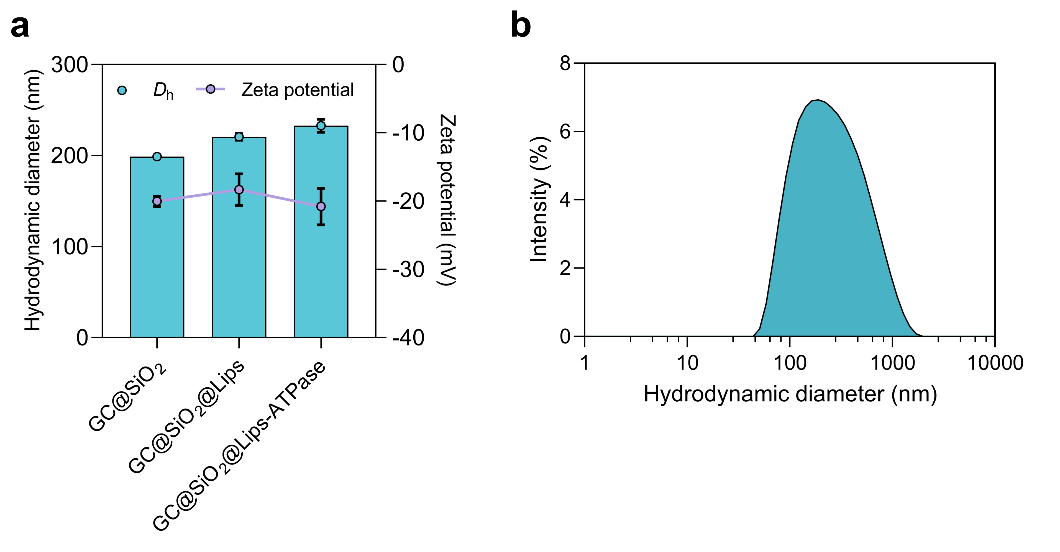


**Figure S15.** (a) *D*_h_ and Zeta potentials of GC@SiO_2_, GC@SiO_2_@Lips, and GC@SiO_2_@Lips-ATPase (n =3). (b) Size distribution of GC@SiO_2_@Lips-ATPase.


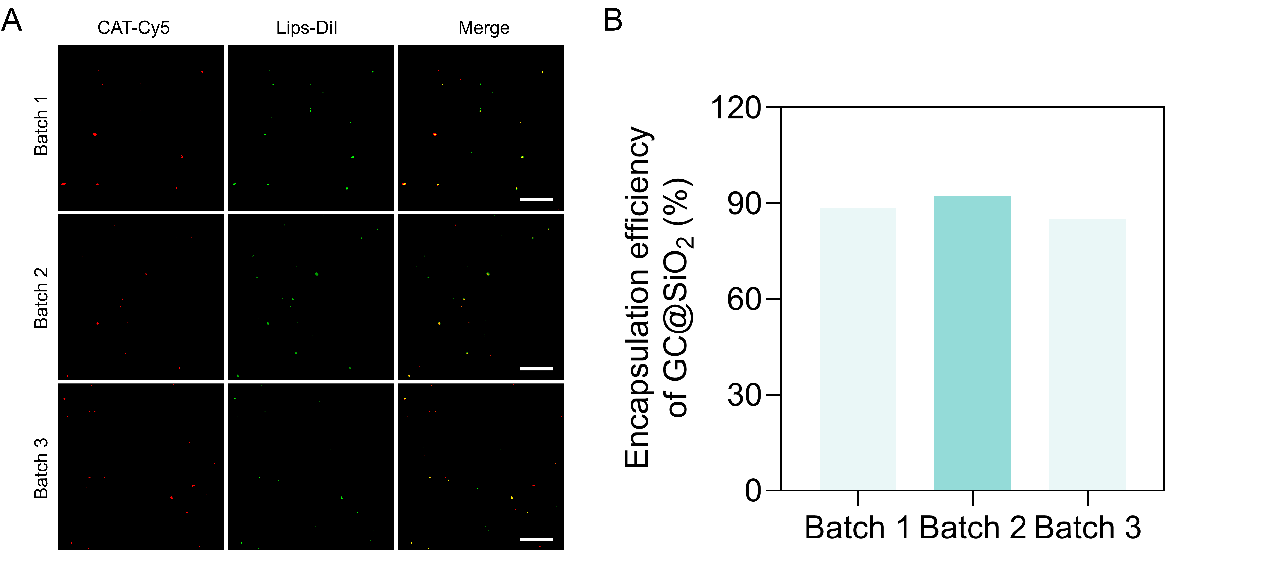


**Figure S16.** (a) CLSM images of GC@SiO_2_@Lips in three independent batches. (b) Encapsulation efficiency of GC@SiO_2_ in liposomes detected based on CLSM images. A representative field of view was randomly selected, and encapsulation efficiency was calculated accordingly.


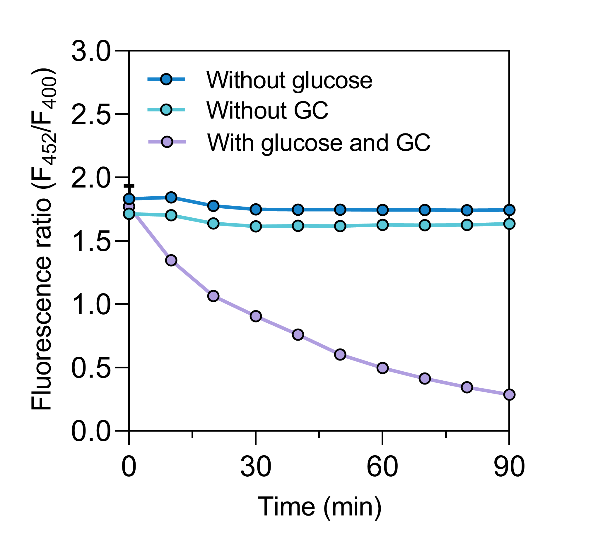


**Figure S17.** Fluorescence ratio of pyranine at 452 nm and 400 nm over time in different enzymatic reaction groups (n = 3).

**
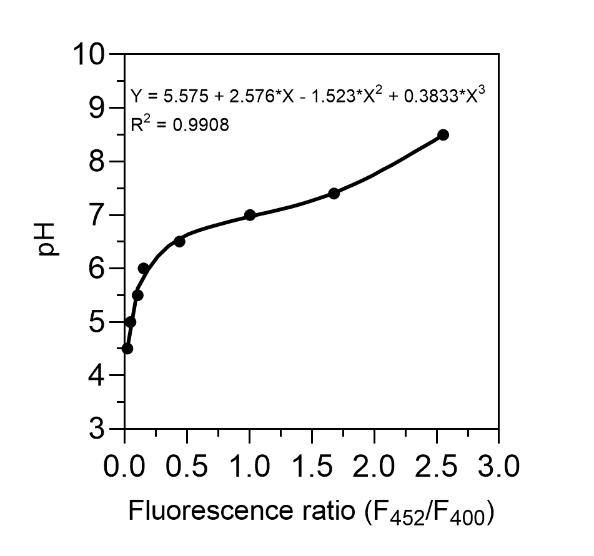
**

**Figure S18.** Standard curve of pH value versus fluorescence ratio of pyranine at 452 nm and 400 nm.

**
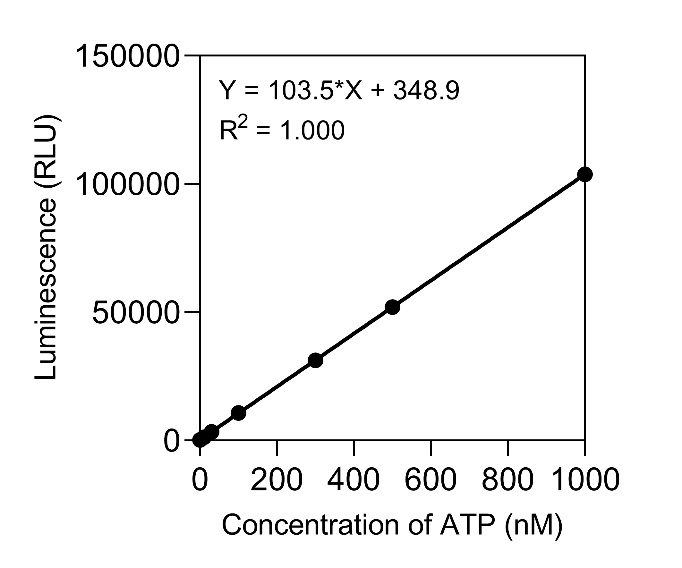
**

**Figure S19.** Standard curve of luminescence intensity versus ATP concentration. The concentration range was 0-1000 nM.


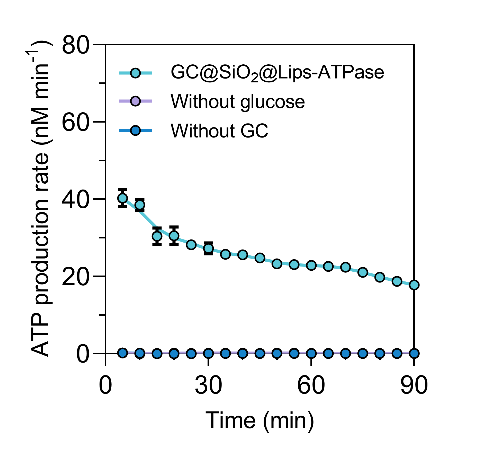


**Figure S20.** Change of ATP production rate with time in different enzymatic reaction groups (n = 3).

**
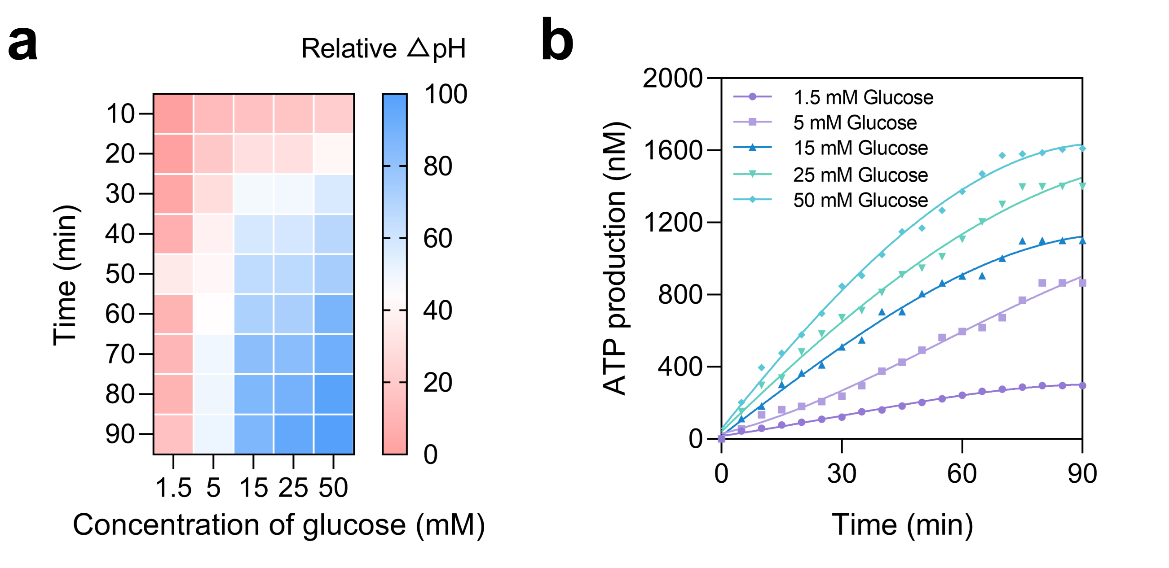
**

**Figure S21.** (a) Time-dependent pH changes and (b) ATP production of GC@SiO_2_@Lips-ATPase under different glucose concentrations.


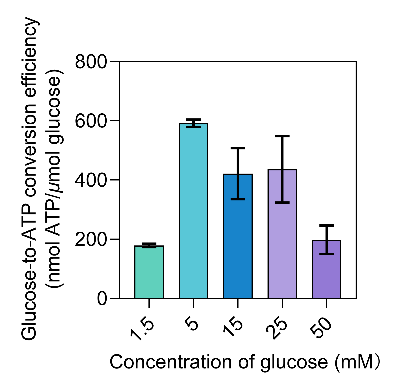


**Figure S22.** Glucose-to-ATP conversion efficiency of GC@SiO2@Lips-ATPase under different glucose concentrations (n = 3).


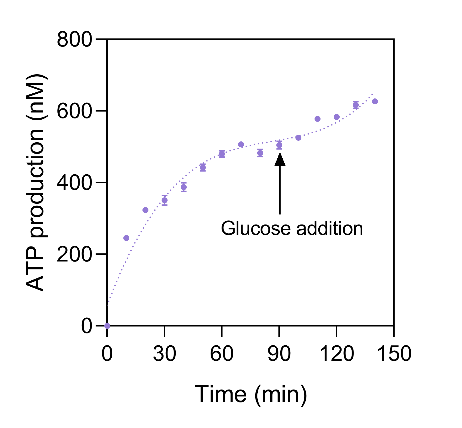


**Figure S23.** The real-time ATP production upon additional glucose supplementation (n = 3).

**
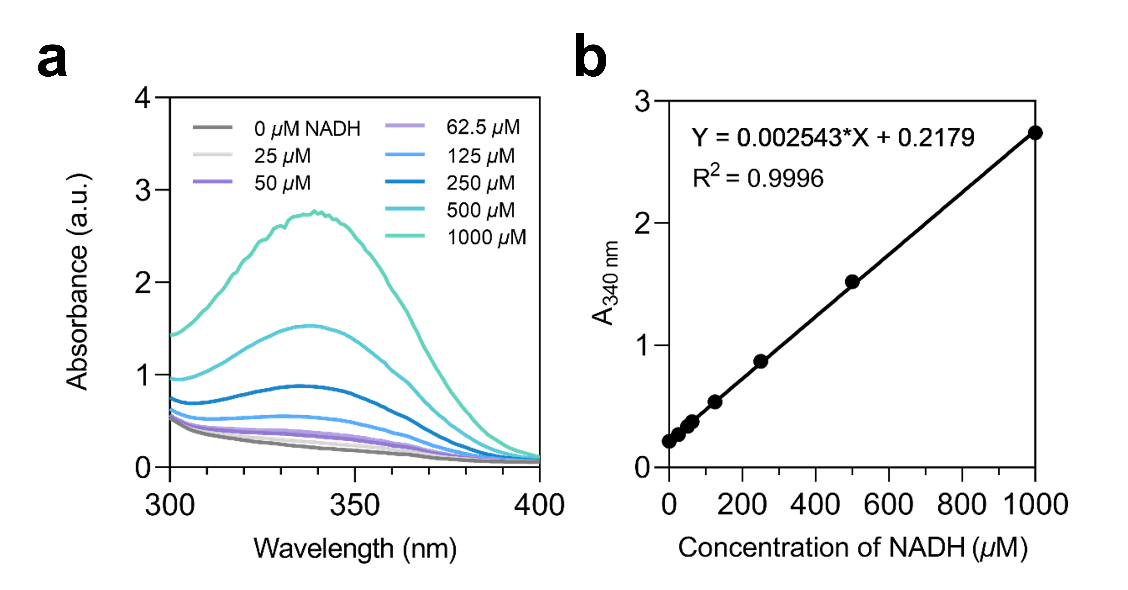
**

**Figure S24.** (a) UV-Vis spectra of NADH at different concentrations. (b) Standard curve of absorbance at 340 nm versus NADH concentration. The concentration range was 0-1000 *μ*M.


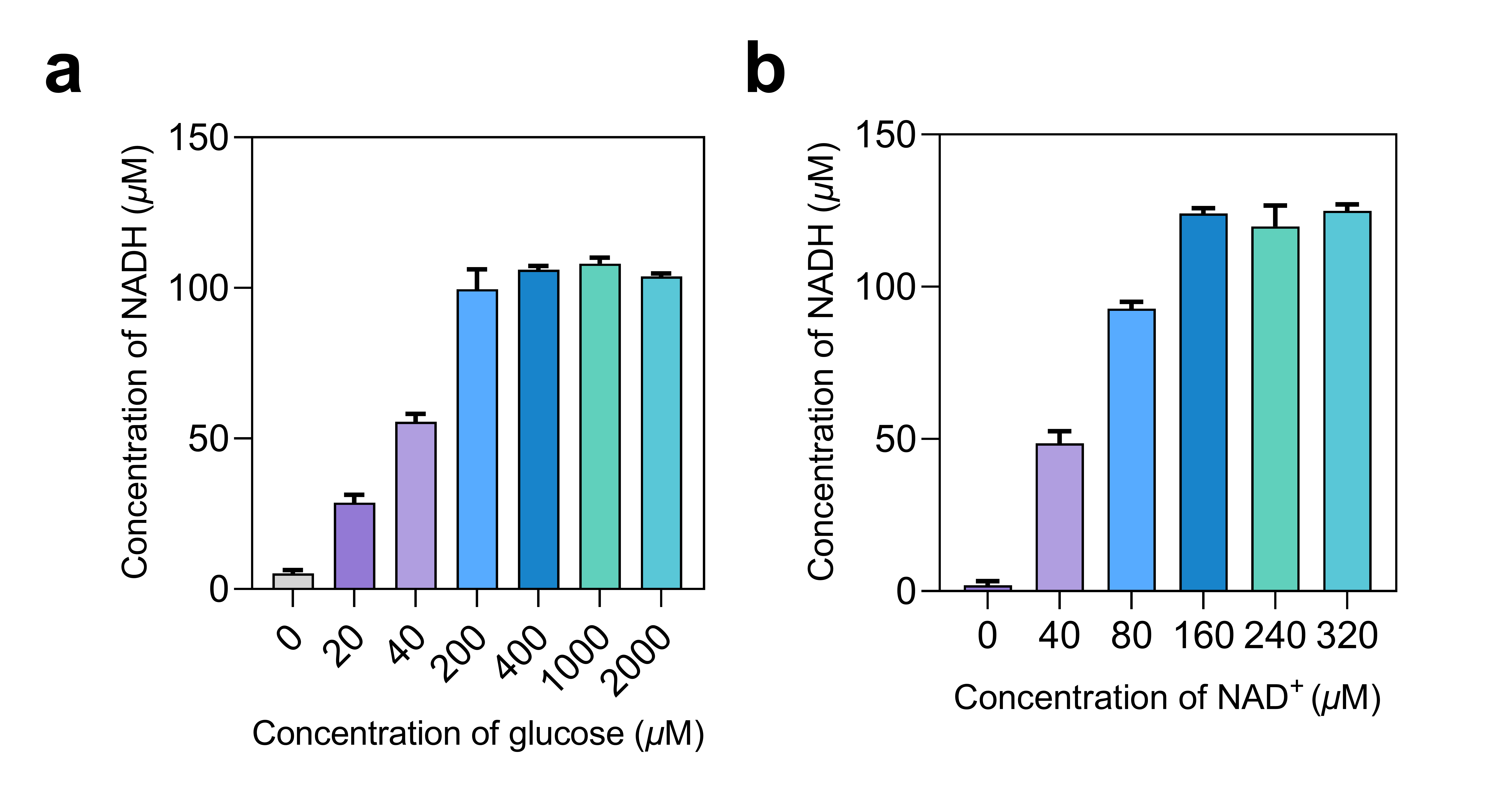


**Figure S25.** NADH production at (a) different glucose concentrations and (b) different NAD^+^ concentrations (n = 3).


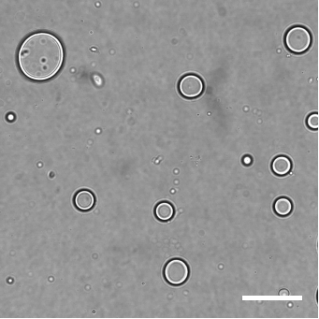


Figure S26. Bright-field CLSM image of GUVs. Scale bar: 20 *μ*m.

**Table S1.** Encapsulation efficiency of GC@SiO_2_

^
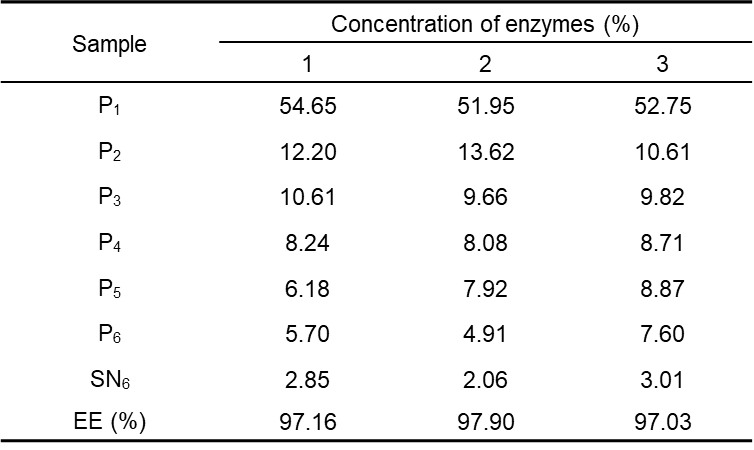
^

P_n_: GC@SiO_2_ collected in the pellets during the centrifugation step (n); SN_6_: supernatant obtained from the final centrifugation step. 1, 2, and 3 represent three independent experiments.

# References

[1] S. Jiang, L. Caire da Silva, T. Ivanov, M. Mottola, K. Landfester, *Angew. Chem.* **2021**, *61*, e202113784.

[2] W. Han, J. Ding, B. Qiao, Y. Yu, H. Sun, D. Crespy, K. Landfester, X. Mao, S. Jiang, *Adv. Mater.* **2025**, *37*, 2415030.

[3] Y. Xu, J. Fei, G. Li, T. Yuan, X. Xu, J. Li, *Angew. Chem.* **2019**, *58*, 5572-5576.

[4] R. Wang, Y. Yu, M. Gai, A. Mateos‐Maroto, S. Morsbach, X. Xia, M. He, J. Fan, X. Peng, K. Landfester, S. Jiang, W. Sun, *Angew. Chem.* **2023**, *62*, e202308761.

[5] B. Yang, S. Li, W. Mu, Z. Wang, X. Han, *Small* **2022**, *19*, 2201305.

[6] Q. Wang, X. Zhang, L. Huang, Z. Zhang, S. Dong, *Angew. Chem.* **2017**, *56*, 16082-16085.

[7] Z. Li, F. Yu, X. Xu, T. Wang, J. Fei, J. Hao, J. Li, *J*. *Am*. *Chem*. *Soc*. **2023**, *145*, 20907-20912.

[8] T. Wang, J. Fei, Z. Dong, F. Yu, J. Li, *Angew. Chem.* **2024**, *63*, e202319116.
